# The pivotal ripening gene *SlDML2* participates in regulating disease resistance in tomato

**DOI:** 10.1111/pbi.14130

**Published:** 2023-07-19

**Authors:** Leilei Zhou, Guangtong Gao, Xiaojing Li, Weihao Wang, Shiping Tian, Guozheng Qin

**Affiliations:** ^1^ Key Laboratory of Plant Resources, Institute of Botany Chinese Academy of Sciences Beijing China; ^2^ China National Botanical Garden Beijing China; ^3^ University of Chinese Academy of Sciences Beijing China

**Keywords:** DNA methylation, SlDML2, defence response, transcriptome reprogramming, *Botrytis cinerea*, tomato

## Abstract

Fruit ripening and disease resistance are two essential biological processes for quality formation and maintenance. DNA methylation, in the form of 5‐methylcytosine (5mC), has been elucidated to modulate fruit ripening, but its role in regulating fruit disease resistance remains poorly understood. In this study, we show that mutation of *SlDML2*, the DNA demethylase gene essential for fruit ripening, affects multiple developmental processes of tomato besides fruit ripening, including seed germination, leaf length and width and flower branching. Intriguingly, loss of SlDML2 function decreased the resistance of tomato fruits against the necrotrophic fungal pathogen *Botrytis cinerea.* Comparative transcriptomic analysis revealed an obvious transcriptome reprogramming caused by *SlDML2* mutation during *B. cinerea* invasion. Among the thousands of differentially expressed genes, *SlβCA3* encoding a β‐carbonic anhydrase and *SlFAD3* encoding a ω‐3 fatty acid desaturase were demonstrated to be transcriptionally activated by SlDML2‐mediated DNA demethylation and positively regulate tomato resistance to *B. cinerea* probably in the same genetic pathway with *SlDML2*. We further show that the pericarp tissue surrounding *B. cinerea* infection exhibited a delay in ripening with singnificant decrease in expression of ripening genes that are targeted by SlDML2 and increase in expression of *SlβCA3* and *SlFAD3*. Taken together, our results uncover an essential layer of gene regulation mediated by DNA methylation upon *B. cinerea* infection and raise the possible that the DNA demethylase gene *SlDML2*, as a multifunctional gene, participates in modulating the trade‐off between fruit ripening and disease resistance.

## Introduction

*Botrytis cinerea*, the pathogenic agent of grey mould, annually causes dramatical agriculture losses due to its widespread and necrotrophic infection characteristics (Williamson *et al*., 2007; Zhang *et al*., 2021). More than 200 economically important crop species are susceptible to *B. cinerea* worldwide (Elad *et al*., 2007; Liu *et al*., 2021). *B. cinerea* generally secretes diverse toxic substances to kill the host cells for acquiring adequate nutrition during the pathogenic process (Cheung *et al*., 2020). Utilization of chemical fungicides represents an efficient strategy at present for controlling fungal diseases, but their overuse harbours potential threats to human health (Jankowska *et al*., 2016). Therefore, it is currently imperative to explore more secure alternatives to effectively control grey mould, and the information concerning the molecular mechanisms of plant disease resistance to *B. cinerea* will provide us new perspectives.

Tomato, one of the most important horticultural crops, is easily accessible for *B. cinerea* invasion, especially the fruits at the postharvest stage that undergo continuous ripening process (Cantu *et al*., 2008, 2009; Prusky *et al*., 2013). The interaction between tomato fruits and *B. cinerea* is generally utilized as a model pathosystem for dissecting the defence mechanisms employed by host cells (Min *et al*., 2020; Silva *et al*., 2023; Wang *et al*., 2017b). Accumulating evidences suggest that tomato cuticles and cell walls are the important physical barriers, and phytoalexins, such as phenolics and flavonoids, act as antimicrobial chemical components to defence *B. cinerea* infection (Xu *et al*., 2022). Phytohormones jasmonic acid (JA) and methyl jasmonate (MJ), the endogenous signal molecules, also play a critical role in regulating tomato resistance to *B. cinerea* disease, partially by inducing the biosynthesis of phytoalexins or activating the transcription of downstream defence genes (Huang *et al*., 2022; Reyes‐Diaz *et al*., 2016; Shu *et al*., 2021). In addition, the resistance of tomato fruits towards *B. cinerea* is dramatically influenced by fruit maturity, and one direct evidence is that ripe red fruits are more susceptible to *B. cinerea* than unripe green fruits (Cantu *et al*., 2009; Prusky *et al*., 2013; Silva *et al*., 2023). This diverse susceptibility is generally thought to be self‐interested, since the fruits need to defence external invasions for surviving until seed maturation, and then spread mature seeds with the aid of enhanced fungal infection (Cantu *et al*., 2009). Thus, fruit ripening and disease resistance are two tightly interconnected biological processes, although the mechanisms underlying their interplays remain elusive. Understanding in the molecular link between fruit ripening and disease resistance has great potential for controlling fungal invasion and maintaining fruit quality.

DNA methylation, in the form of 5‐methylcytosine (5mC), is a broad‐spectrum epigenetic modification, mainly occurring in sequences of gene promoters, gene bodies and transposable elements (Bartels *et al*., 2018). DNA methylation modification generally facilitates transcriptional inactivation through remodelling chromatin structure or regulating the accessibility of transcriptional regulators to associated gene sequences (Zhang *et al*., 2018). DNA methylation is subjected to the synergistic action of DNA methyltransferases and demethylases, thereby exhibits a dynamic status *in vivo* and participates in the regulation of many important biological processes, including plant stress resistance, genome management, and developmental process (Bartels *et al*., 2018; Chen *et al*., 2020; Tang *et al*., 2020). More recently, DNA methylation was also demonstrated to regulate plant–pathogen interactions in agricultural and horticultural crops, including *Magnaporthe oryzae*‐infected rice, *Fusarium graminearum*‐infected maize, *Xanthomonas phaseoli*‐infected cassava and *Fusarium. oxysporum*‐infected banana (Deng *et al*., 2017; Luo *et al*., 2016; Tirnaz and Batley, 2019; Veley *et al*., 2023; Wang *et al*., 2017a). In addition, DNA methylation functionally modulates the disease resistance of woody plant mulberry towards *B. cinerea*, and the DNA methyltransferase gene *MnMET1* plays a negative regulatory role in *B. cinerea* invasion through impeding the transcription of defence genes by DNA hypermethylation (Xin *et al*., 2021). However, whether DNA methylation participates in regulating the disease resistance of horticultural crops including tomato to *B. cinerea* has not been defined. Moreover, the trade‐off between DNA methylation‐mediated disease resistance and fruit ripening remains to be determined.

In tomato, there are four DEMETER‐like (DML) DNA demethylase genes, i.e. *SlDML1‐SlDML4* (Liu *et al*., 2015). *SlDML2*, the homologue of Arabidopsis *Repressor of Silencing 1* (*AtROS1*) encoding a 5mC DNA glycosylase, has been well defined as a pivotal ripening gene for its significant functions in facilitating the expression of ripening‐related genes through active DNA demethylation (Gao *et al*., 2022; Lang *et al*., 2017; Li *et al*., 2020; Zhou *et al*., 2019). In the present study, we investigated the functional role of *SlDML2* in the pathogenic process of *B. cinerea* and found that fruits and leaves of the *sldml2* mutants generated by CRISPR/Cas9 gene editing system exhibited increased susceptibility to *B. cinerea* compared with those of the wild‐type. The comparative transcriptomic analysis between wild‐type and *sldml2* mutant fruits after *B. cinerea* inoculation revealed that *SlDML2* is required for the expression of plenty of defence‐related genes, including the CO_2_‐binding protein gene *SlβCA3* encoding a β‐carbonic anhydrase and the JA biosynthetic gene *SlFAD3* encoding a ω‐3 fatty acid desaturase (Domínguez *et al*., 2010; Hu *et al*., 2021; Yu *et al*., 2009). We provided evidences that both *SlβCA3* and *SlFAD3* undergo SlDML2‐mediated transcriptional activation and play a positive role against *B. cinerea* invasion probably in the same genetic pathway with *SlDML2*. Furthermore, we found that *B. cinerea* infection causes a delay in fruit ripening in the pericarp tissue surrounding disease regions, accompanied by the decreased expression of ripening genes targeted by SlDML2 and the increased expression of *SlβCA3 and SlFAD3*. Our findings uncover the dual roles of SlDML2 in regulating fruit ripening and defence response to fungal pathogen and highlight the possible function of SlDML2 as a molecular link between ripening and disease resistance in fruit.

## Results

### SlDML2 modulates multiple developmental processes besides fruit ripening

**Figure 1:**
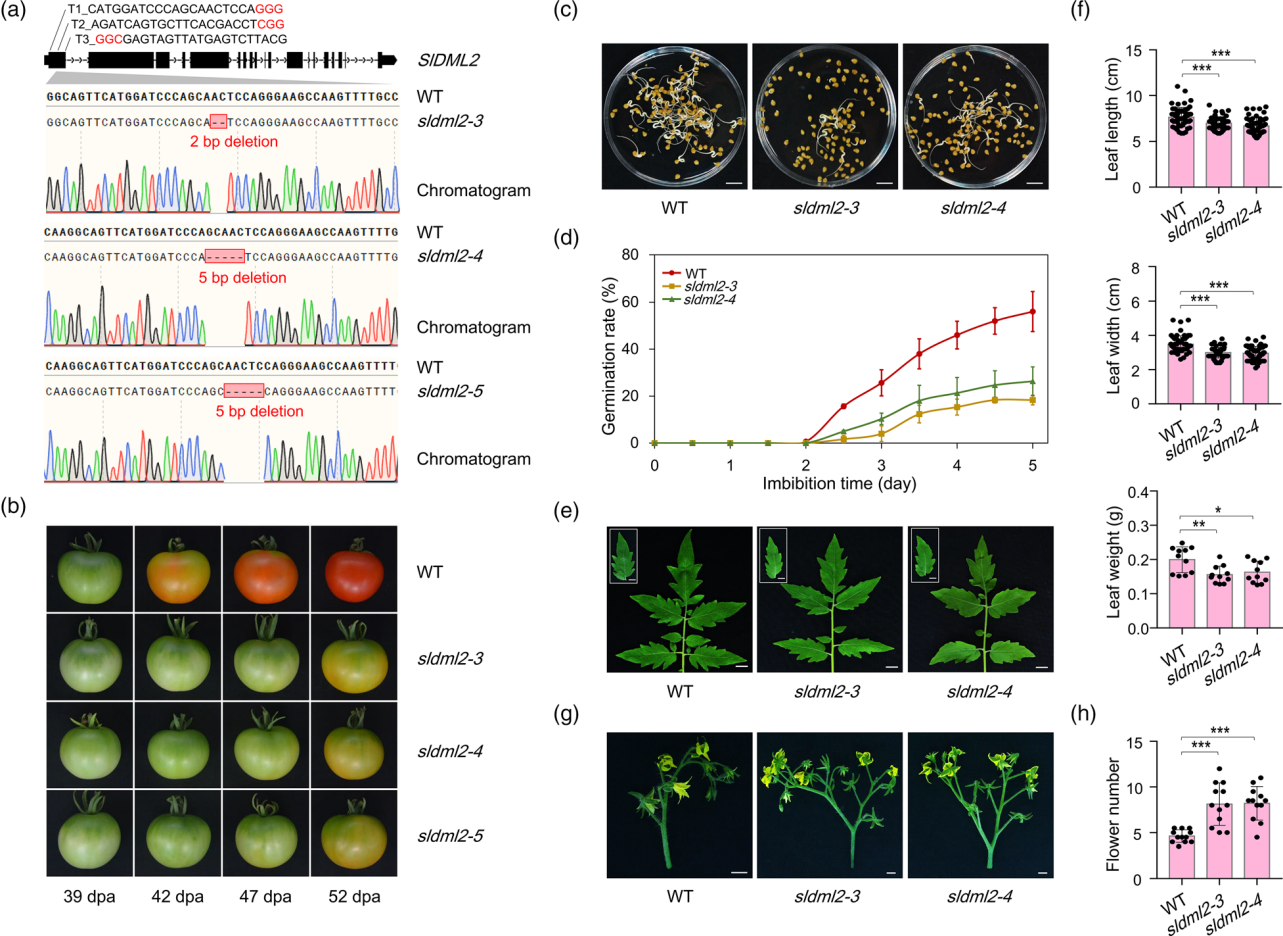
SlDML2 participates in modulating multiple developmental processes. (a) Genotyping of *sldml2‐3*, *sldml2‐4*, and *sldml2‐5* mutants generated by CRISPR/Cas9‐mediated gene editing. Three single guide RNAs (sgRNAs) containing different target sequences (T1, T2, and T3) were designed to specifically target the first exon of *SlDML2*. The red letters indicate the protospacer adjacent motif (PAM). The *sldml2‐3* mutant contains a homozygous 2‐bp deletion, and the *sldml2‐4* and *sldml2‐5* mutants contain a homozygous 5‐bp deletion caused by target T1. (b) Phenotypes of fruit ripening in *sldml2* mutants. Representative photographs of the wild‐type (WT), *sldml2‐3*, *sldml2‐4*, and *sldml2‐5* fruits at 39, 42, 47, and 52 dpa are shown. dpa, days post‐anthesis. (c) Representative photographs and (d) germination rates of the WT, *sldml2‐3*, and *sldml2‐4* seeds. For each germination experiment, one‐hundred mature seeds were cultured with deionized water under long‐day conditions (16 h light/8 h dark, 25 °C). The gemmiparous seeds were photographed at the fourth day after imbibition. Data are presented as mean ± standard deviation (*n* = 3). (e) Representative photographs and (f) length, width, and weight of the WT, *sldml2‐3*, and *sldml2‐4* leaves. Six‐week‐old leaves were collected, photographed, and measured. (g) Representative photographs and (h) flower number of the WT, *sldml2‐3*, and *sldml2‐4* inflorescences. The first inflorescences were collected and photographed, and the flower number in each inflorescence was measured. In (c), (e), and (g), scale bar = 1 cm. In (f and h), asterisks indicate significant differences (*P < 0.05, **P < 0.01, ***P < 0.001; Student's *t* test).

Tomato DNA demethylase SlDML2 has been considered primarily to modulate fruit ripening through activating 5mC demethylation in numerous ripening genes (Lang *et al*., 2017; Liu *et al*., 2015). Recently, emerging evidences from DNA methylomes indicate that SlDML2 potentially targets thousands of genes relevant to distinct biological processes, rather than merely those of fruit ripening (Gao *et al*., 2022; Lang *et al*., 2017). To further explore the physiological roles of SlDML2, we first generated its loss‐of‐function mutant by CRISPR/Cas9‐mediated gene editing. Three single guide RNAs (sgRNAs) with different target sequences (i.e., T1, T2 and T3) were designed to specifically target the first exon of *SlDML2* (Figure 1a). The sgRNAs were introduced into the binary vector pYLCRISPR/Cas9Pubi‐H that contains a Cas9 expression cassette, and then transformed into leaf explants of wild‐type tomato (*Solanum lycopersicum* cv. Ailsa Craig) by using *Agrobacterium* infection (Fillatti *et al*., 1987). Following the standard tissue culture, a total of six transgenic lines were obtained. Among them, three homozygous mutant lines that carry 2‐bp or 5‐bp deletion were identified through genotyping mediated by direct sequencing of PCR products (Figure 1a). Both the 2‐bp and 5‐bp deletion mutations, which were predicted to cause premature stop codons in the first exon of *SlDML2*, occurred in the DNA sequence targeted by the first sgRNA. The three homozygous mutants, namely *sldml2‐3*, *sldml2‐4* and *sldml2‐5*, were predicted to produce short peptides with a length of 30–40 amino acids, respectively (Figure S1).

To determine the ripening phenotype, fruits of the wild‐type and *sldml2* mutants (*sldml2‐3*, *sldml2‐4* and *sldml2‐5*) at the second generation were harvested at 39, 42, 47 and 52 days post‐anthesis (dpa), when the wild‐type fruits reach to mature green (MG), breaker (Br), orange ripe (OR) and red ripe (RR) stages, respectively. Compared to the wild‐type, the *sldml2* mutants showed a similar and obvious delay in ripening, especially at 52 dpa, when fruits of the wild‐type exhibited a homogenous red surface, whereas those of the *sldml2* mutants were only just starting to change colour (Figure 1b). This ripening phenotype is consistent with that of previously reported *sldml2* mutants in Micro‐Tom background (Lang *et al*., 2017), both indicating that SlDML2 is necessary for normal fruit ripening. Fruits of the *sldml2* mutants displayed a homogenous red colour at approximately 90 dpa, similar to those of the wild‐type (Figure S2), suggesting that fruits of the *sldml2* mutants harbour the capacity to reach red ripe.

We next sought to determine whether SlDML2 functions in other developmental processes besides fruit ripening. As expected, tomato seeds, leaves and flowers exhibited abnormal developmental phenotypes under the disruption of *SlDML2* gene. The germination rate of seeds from the *sldml2* mutants decreased obviously under long‐day conditions (16 h light/8 h dark, 25 °C) compared to those from the wild‐type (Figure 1c,d), although no significant difference was detected in seed length, width and weight (Figure S3a,b). The leaves of *sldml2* mutants were smaller than those of the wild‐type with shorter length and width, and lighter weight (Figure 1e,f). The flower branching of *sldml2* mutants had more floral organs than those of the wild‐type (Figure 1g,h), indicating a floriferous phenomenon caused by *SlDML2* mutation. Quantitative RT‐PCR analysis showed that *SlDML2* gene was highly expressed in tomato seeds, leaves and flowers besides the fruits (Figure S4). These results revealed the functional diversity of SlDML2 in modulating multiple developmental processes in tomato.

### SlDML2 positively regulates resistance of tomato fruits to fungal pathogens *B. cinerea*

**Figure 2:**
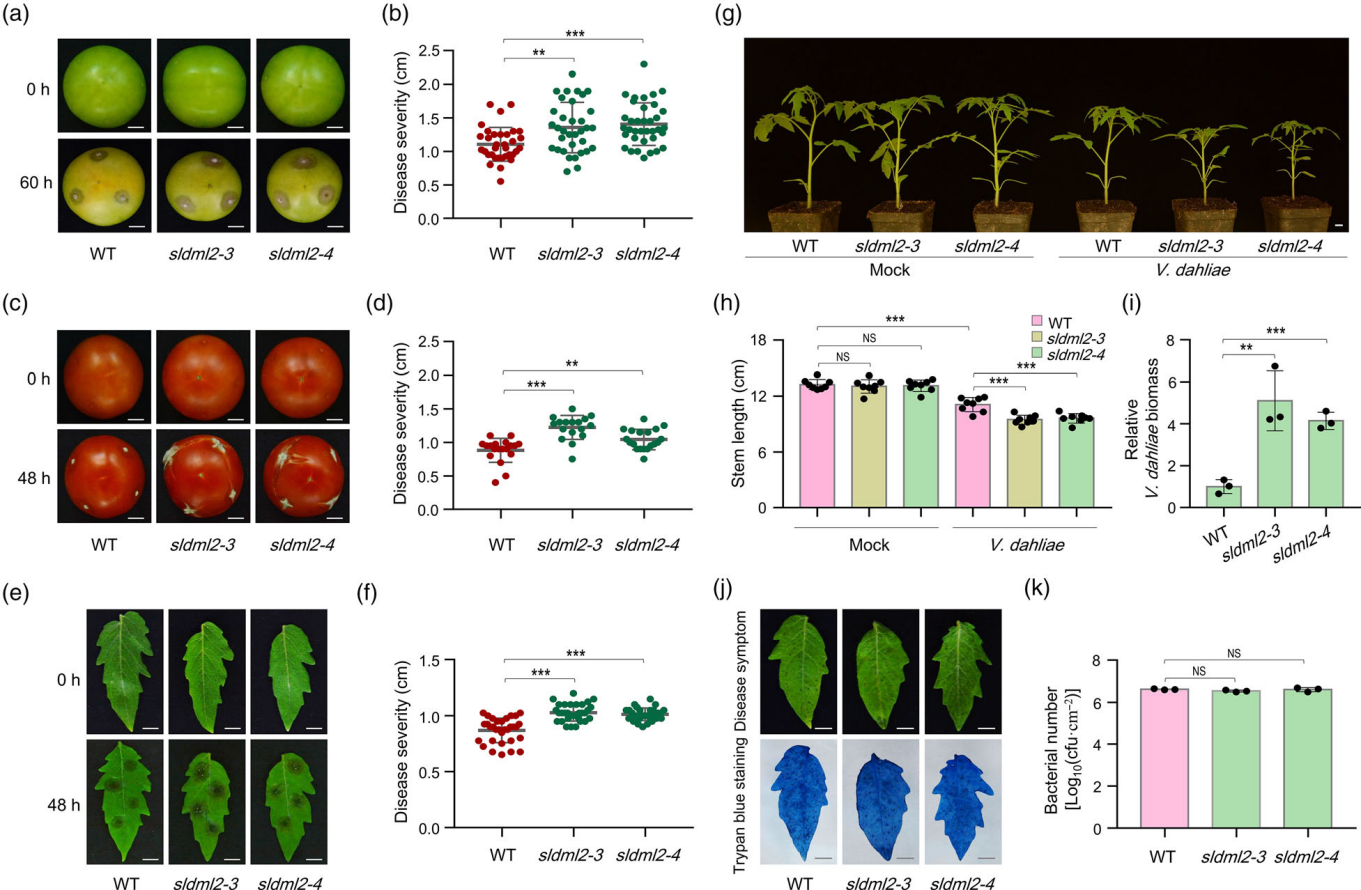
The *sldml2* mutants exhibited decreased resistance to fungal pathogens *B. cinerea* and *V. dahliae*. (a) Disease symptoms and (b) lesion diameters on the detached wild‐type (WT), *sldml2‐3*, and *sldml2‐4* fruits inoculated with *B. cinerea* at 39 days post‐anthesis (dpa). The disease symptom was observed after 60 h. (c) Disease symptoms and (d) lesion diameters on the detached WT, *sldml2‐3*, and *sldml2‐4* fruits inoculated with *B. cinerea* at 90 dpa. The disease symptom was observed after 48 h. (e) Disease symptoms and (f) lesion diameters on 4‐week‐old detached leaves of WT, *sldml2‐3*, and *sldml2‐4* inoculated with *B. cinerea*. The disease symptom was observed after 48 h. (g) Representative photographs and (h) stem lengths of the WT, *sldml2‐3*, and *sldml2‐4* seedlings that were either inoculated with *V. dahliae* or mock‐inoculated for 18 days. (i) Relative *V. dahliae* biomass in stems of the WT, *sldml2‐3*, and *sldml2‐4* seedlings that were inoculated with *V. dahliae* for 18 days. The amounts of *V. dahliae GADPH* gene were determined by quantitative RT‐PCR analysis, and the tomato *RuBisCo* gene was used as an internal control. (j) Disease symptoms and trypan blue staining and (k) bacterial biomass in WT, *sldml2‐3*, and *sldml2‐4* leaves that were inoculated with *Pst* DC3000 for 3 days. In (a), (c), (e), (g), and (j), scale bar = 1 cm. In (b, d, f, h, i and k), asterisks indicate significant differences (**P < 0.01, ***P < 0.001; Student's *t* test). NS, no significance.

Having observed the developmental phenotypes, we next investigated whether SlDML2 participates in modulating tomato resistance to *B. cinerea* invasion, which is generally known to cause serious yield and quality declines in horticultural crops including tomato (Blanco‐Ulate *et al*., 2013; Tian *et al*., 2016). Fruits of the wild‐type and *sldml2* mutants harvested at 39 and 90 dpa were inoculated with *B. cinerea* stain B05.10, and the lesion diameters were measured at the second to third day post‐inoculation (dpi) when obvious soft rot symptoms appeared. It was shown that green and red fruits of the *sldml2* mutants (at 39 and 90 dpa, respectively) exhibited significantly larger lesion diameters than the wild‐type (Figure 2a–d). We concurrently detected the leaf susceptibility to *B. cinerea* infection and found that leaves of the *sldml2* mutants also displayed larger lesion diameters by comparison with the wild‐type (Figure 2e,f). These results indicate that the disease resistance was impaired in the *sldml2* mutants, suggesting that SlDML2 positively regulates the resistance response of tomato to *B. cinerea*.

We next asked, besides the necrotrophic pathogen *B. cinerea*, whether SlDML2 regulates tomato resistance to biotrophic pathogens, such as *Verticillium dahliae* and *Pseudomonas syringae* pv. *tomato* (*Pst*) DC3000. *V. dahliae* is a soil‐borne fungal pathogen responsible for severe losses of biomass, yield and quality in plenty of economically important crops including tomato and cotton (Zhang *et al*., 2023), while *P. syringae* represents a model biotrophic bacterial pathogen that has been extensively investigated in tomato and Arabidopsis (Xin and He, 2013). Three‐week‐old tomato seedlings of wild‐type and *sldml2* mutants that display non‐differential stem lengths were inoculated with *V. dahliae* and the disease response was observed at 18 days post‐inoculation (dpi) (Figure S5a,b). Compared to the mock group, the *V. dahliae*‐infected tomato seedlings exhibited a dwarf phenotype in both wild‐type and *sldml2* mutants, implying an obvious growth inhibition caused by *V. dahliae* invasion (Figure 2g,h). Importantly, the seedlings of *V. dahliae*‐infected *sldml2* mutants displayed significantly shorter stem length compared to the *V. dahliae*‐infected wild‐type, indicating a more serious growth inhibition after *V. dahliae* invasion (Figure 2g,h). Detection of *V. dahliae* biomass by relative quantification of *V. dahliae GADPH* gene in tomato stems revealed an obvious increase in *GADPH* expression (~4–6‐fold) in the *sldml2* mutants compared to the wild‐type (Figure 2i). These results support that SlDML2 also positively regulates tomato resistance to the biotrophic pathogen *V. dahliae*.

To explore whether SlDML2 regulates the resistance of tomato to *Pst* DC3000, 4‐week‐old leaves of the wild‐type and *sldml2* mutants were inoculated with *Pst* DC3000, and the disease response was measured at 3 dpi. Similar disease symptom and cell death were observed in the wild‐type and *sldml2* mutant leaves (Figure 2j). Bacterial growth assay demonstrated no significant difference in bacterial number between leaves of the wild‐type and *sldml2* mutants (Figure 2k), suggesting that SlDML2 is dispensable for tomato resistance to the biotrophic bacterial pathogen *Pst* DC3000. Together, these data suggest that SlDML2 confers resistance of tomato to the necrotrophic fungal pathogen *B. cinerea* and biotrophic fungal pathogen *V. dahliae*, but not to the biotrophic bacterial pathogen *Pst* DC3000.

### *SlDML2* mutation causes transcriptome reprogramming in tomato fruits during *B. cinerea* infection

**Figure 3:**
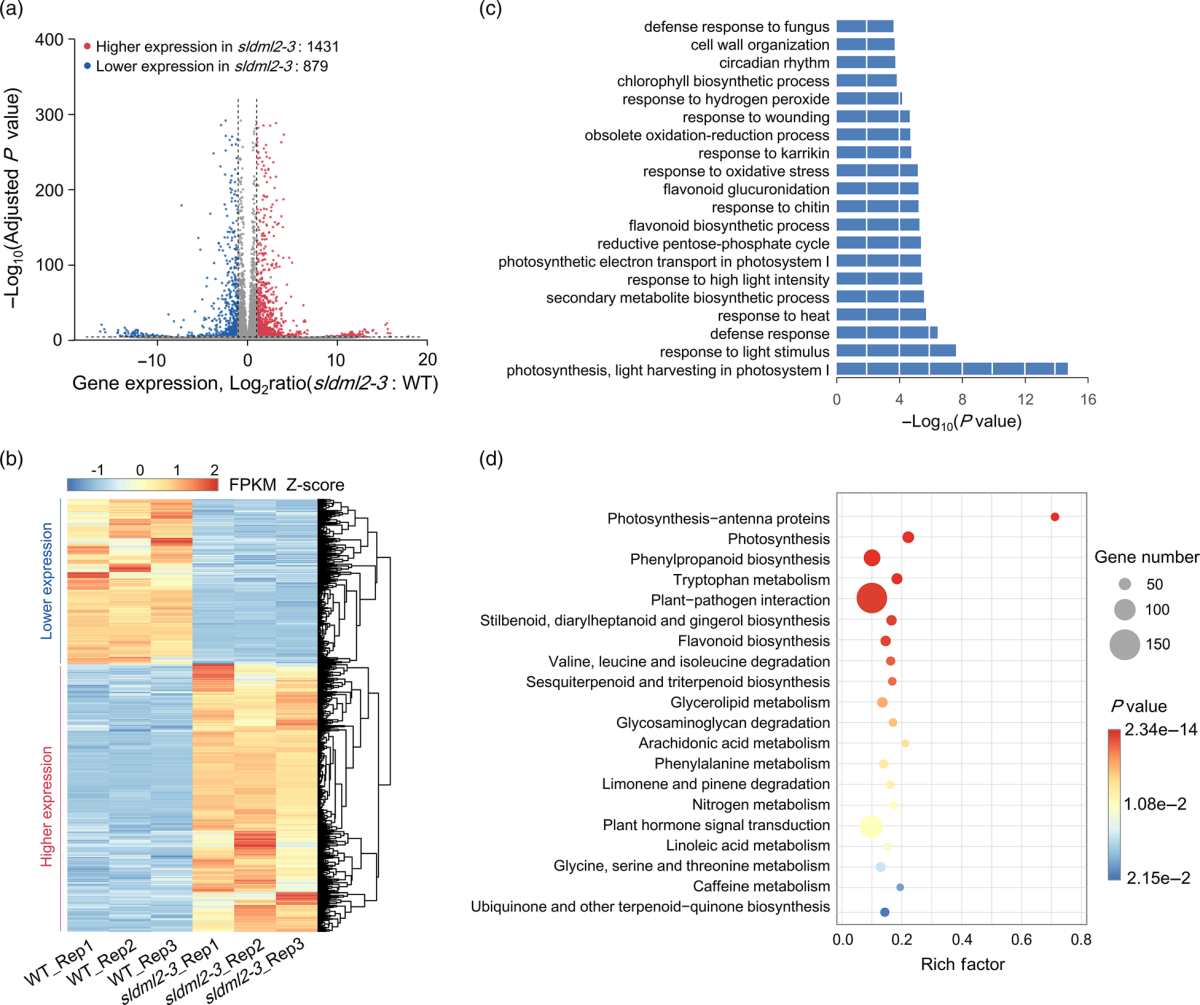
*SlDML2* mutation causes transcriptome reprogramming during *B. cinerea* infection. (a) Volcano plot showing up‐regulated (red) and down‐regulated (blue) genes in the *sldml2‐3* mutant compared to the wild‐type (WT). (b) Heat map of differentially expressed genes in the *sldml2‐3* mutant compared with the wild‐type. FPKM, fragments per kilobase of exon per million mapped fragments. (c) Gene ontology (GO) enrichment for the differentially expressed genes in the *sldml2‐3* mutant compared with the WT. The top 20 catalogues in biological process with the most significant *P* value were shown. (d) KEGG analysis for the differentially expressed genes in the *sldml2‐3* mutant compared with the WT. The top 20 pathways with the most significant *P* value were shown. The differentially expressed genes were analysed by three independent RNA‐seq experiments in fruits of the WT and *sldml2‐3* mutant after *B. cinerea* inoculation for 48 h.

To decipher the molecular basis underlying SlDML2‐mediated defence response of tomato fruits to *B. cinerea*, a comparative transcriptomic analysis was performed using *B. cinerea*‐infected tomato fruits of the *sldml2‐3* mutant and wild‐type at 39 dpa. High Pearson correlation coefficients were observed between biological replicates, representing the excellent repeatability (Figure S6). A total of 2310 differentially expressed genes (DEGs; fold change ≥2 and adjusted *P* value <0.05) were identified, of which 1431 were up‐regulated and 879 were down‐regulated in fruits of the *sldml2‐3* mutant compared to the wild‐type (Figure 3a,b; Tables S1 and S2). This indicates an obvious transcriptome reprogramming caused by *SlDML2* mutation in the process of *B. cinerea* infection.

Gene ontology (GO) enrichment analysis revealed that those DEGs were highly enriched in items related to defence response to biotic and abiotic stresses (Figure 3c; Table S3). Importantly, several items associated with fungal pathogen infection are included in the top 20 catalogues with the most significant *P* value, including ‘defense response to fungus’, ‘response to chitin’, ‘response to wounding’, ‘response to oxidative stress’ and ‘cell wall organization’ (Figure 3c). KEGG analysis revealed that the DEGs are involved in multiple pathways, in addition to those closely associated with metabolism (Figure 3d). Among them, the ‘plant–pathogen interaction’ pathway contains the largest number of genes with high significance (Figure 3d). These data uncovered the involvement of SlDML2 in the transcriptional regulation of defence genes related to tomato disease resistance.

### SlDML2‐mediated DNA demethylation facilitates *SlβCA3* expression

We subsequently focused on genes with significantly lower expression level in *B. cinerea*‐infected *sldml2‐3* fruits for the reason that loss‐of‐function of SlDML2 is likely to induce DNA hypermethylation, which is generally thought to cause transcriptional inactivation. Comparison of those down‐regulated genes (Table S2) with genes containing hypermethylated DMR (differentially methylated region) identified in the *sldml2* mutants (Lang *et al*., 2017) revealed that, among the 879 down‐regulated genes, 528 (~60%) were hypermethylated under *SlDML2* disruption (Table S4), suggesting a critical transcription regulation of those genes by SlDML2‐mediated DNA demethylation.

**Figure 4:**
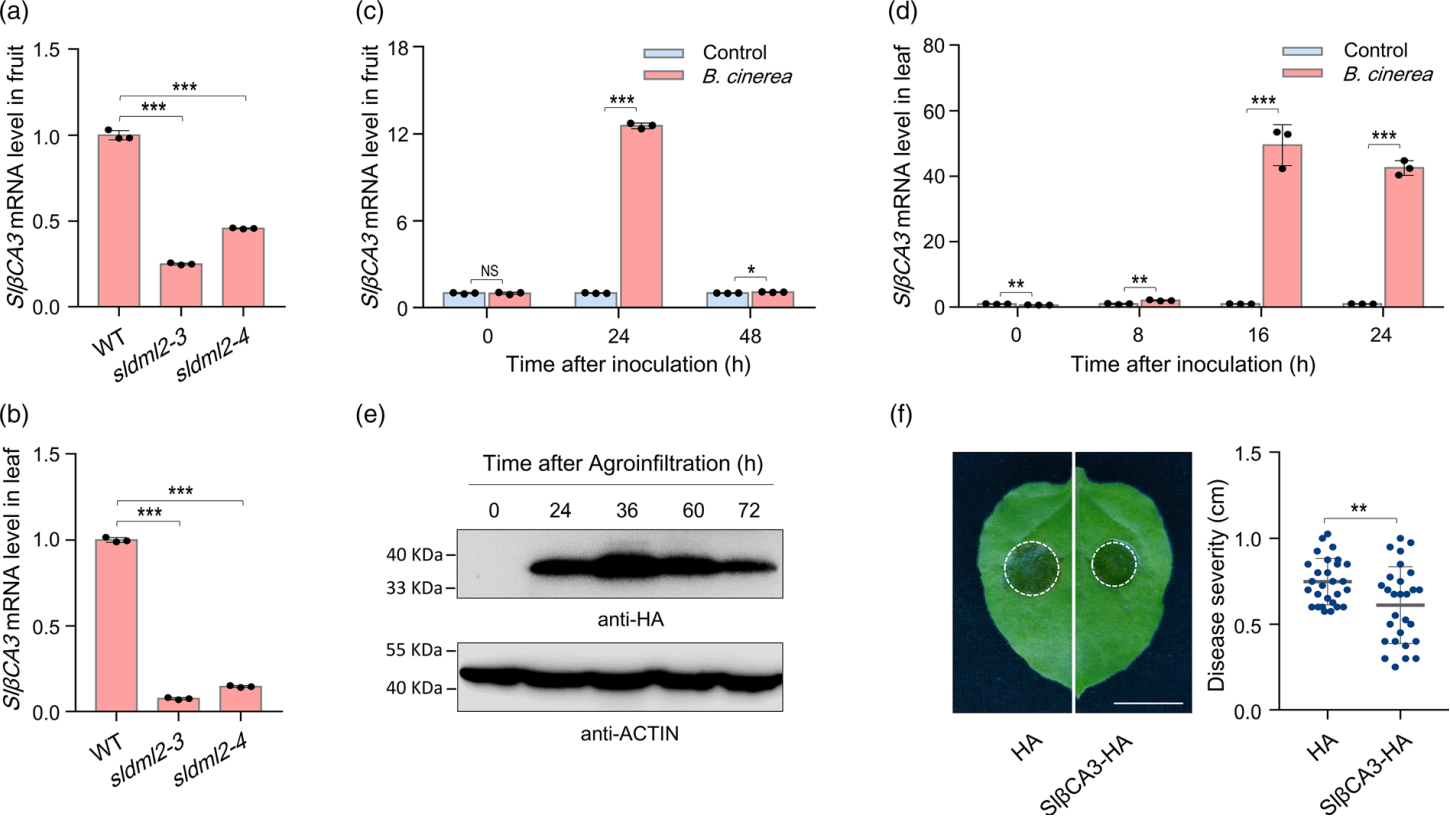
*SlβCA3* is involved in the defence response to *B. cinerea*. (a) Transcription levels of the tomato β‐type carbonic anhydrase gene *SlβCA3* in fruits of the wild‐type (WT) and *sldml2* mutants after *B. cinerea* inoculation for 48 h. (b) Transcription levels of the *SlβCA3* gene in leaves of the WT and *sldml2* mutants after *B. cinerea* inoculation for 48 h. (c) Transcription levels of the *SlβCA3* gene in WT fruits with or without *B. cinerea* inoculation at the indicated time. (d) Transcription levels of the *SlβCA3* gene in WT leaves with or without *B. cinerea* inoculation at the indicated time. In (a–d), the *SlβCA3* transcription levels were determined by quantitative RT‐PCR analysis, and the tomato *SlUBI3* gene was used as an internal control. Asterisks indicate significant differences (*P < 0.05, **P < 0.01, ***P < 0.001; Student's *t* test). NS, no significance. (e) Transient expression of SlβCA3‐HA protein in *N. benthamiana* leaves. Total protein was extracted at indicated time after agroinfiltration and then submitted to immunoblot with anti‐HA antibody. Equal loading was confirmed by using the tomato Actin as an internal control. (f) *B. cinerea* disease symptoms and lesion diameters on detached *N. benthamiana* leaves with or without the co‐expression of SlβCA3‐HA. Asterisks indicate significant differences (**P < 0.01; Student's *t* test). Scale bar = 1 cm.

Among the down‐regulated genes identified in the *sldml2‐3* mutant during *B. cinerea* invasion, a gene encoding the β‐type carbonic anhydrase, namely *SlβCA3* (Solyc02g067750) (Table S2), was previously reported to positively regulate tomato resistance to the virulent pathogen *Pst* DC3000 (Hu *et al*., 2021). *SlβCA3* gene exhibited lower transcription level in fruits or leaves of the *sldml2‐3* and *sldml2‐4* mutants than the wild‐type after *B. cinerea* inoculation for 2 days, as revealed by quantitative RT‐PCR (Figure 4a,b). The transcription level of *SlβCA3* was also obviously decreased in fruits of the *sldml2* mutants under normal developmental process (Figure S7a). These results indicate that SlDML2 positively regulates *SlβCA3* expression.

We next investigated whether SlβCA3 functions in modulating *B. cinerea* disease resistance*. SlβCA3* expression was strongly induced in fruits and leaves of the wild‐type tomato following inoculation with *B. cinerea* (Figure 4c,d), suggesting it is a *B. cinerea*‐responsive gene. We then transiently expressed SlβCA3 protein in *Nicotiana benthamiana* leaves and inoculated *B. cinerea* at the 36 h post‐agroinfiltration when the protein was substantially expressed (Figure 4e). Analysis of the disease symptoms at the second day post‐inoculation showed that the lesion diameters were significantly decreased upon the expression of SlβCA3 protein (Figure 4f), indicating that SlβCA3 has the ability to restrict *B. cinerea* invasion. We also evaluated the function of SlβCA3 in modulating resistance to *V. dahliae* and *Pst* DC3000 and found that SlβCA3 showed no effect on *V. dahliae* (Figure S8), but conferred resistance to *Pst* DC3000 (Figure S9a) as previously reported (Hu *et al*., 2021).

**Figure 5:**
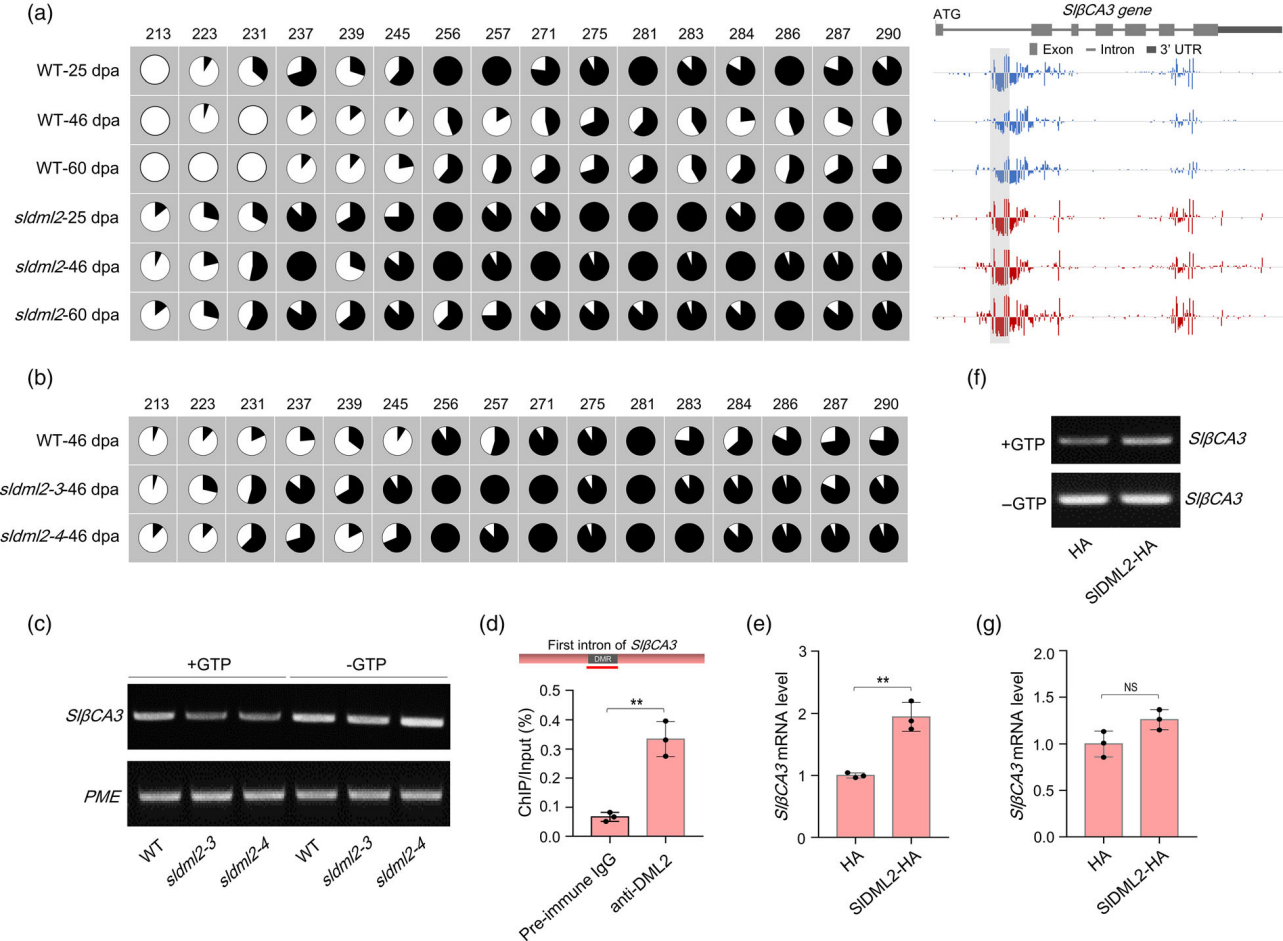
*SlβCA3* is transcriptionally regulated by SlDML2‐mediated DNA demethylation. (a) DNA methylation (5mC) level of the *SlβCA3* gene body in fruits of the wild‐type (WT) and *sldml2* mutant at indicated developmental stages. The 5mC level was analysed by using the published DNA methylome database (Lang *et al*., 2017). Each vertical bar represents an 5mC and the bar height indicates 5mC level. The differentially methylated region (DMR) in the first intron of *SlβCA3* gene was indicated by a shadow box. 5mC levels of representative cytosines in the DMR were shown with pie charts, and the numbers indicate the positions relative to the start codon. dpa, days post‐anthesis. (b) Bisulphite sequencing showing 5mC levels of the representative cytosines shown in (a) in fruits of the wild‐type and *sldml2* mutants (*sldml2‐3* and *sldml2‐4*) at 46 dpa. (c) 5mC level of the DMR of *SlβCA3* gene in fruits of the WT and *sldml2* mutants after *B. cinerea* inoculation for 48 h. (d) ChIP‐qPCR assay showing that SlDML2 binds to the DMR in the first intron of *SlBCA3* gene. The wild‐type fruits at 42 dpa were subjected to the immunoprecipitation with the anti‐SlDML2 polyclonal antibody, and the pre‐immune IgG was used as a control. The first intron structure of *SlβCA3* is shown, and the red line indicates the region for PCR. (e) Transcription levels of the *SlβCA3* gene in *N. benthamiana* leaves with or without the co‐expression of SlDML2‐HA. The sequence of *SlβCA3* gene body containing all exons and introns was cloned into the dual‐luciferase reporter vector for transcription activity assay under the driving of its native promoter. The renilla luciferase gene *RLUC* was used as an internal control. (f) 5mC level of the DMR of *SlβCA3* gene in *N. benthamiana* leaves with or without the co‐expression of SlDML2‐HA. (g) Transcription levels of the mutated *SlβCA3* gene under the drive of its native promoter in *N. benthamiana* leaves with or without the co‐expression of SlDML2‐HA. The representative cytosines shown in (a) were mutated to adenine or thymine. NS, no significance. In (c) and (f), 5mC level was revealed by McrBC‐PCR assay. A total of 0.4 μg genomic DNA was digested by McrBC enzyme with GTP (+GTP), or without GTP (−GTP) as a negative control. An unmethylated region in the promoter of tomato *PME* gene was used as an internal control. In (d and e), asterisks indicate significant differences (**P < 0.01; Student's *t* test).

According to the published DNA methylomes in *sldml2* mutants (Lang *et al*., 2017), no DMR exists in the *SlβCA3* promoter region (~2000 bp upstream of the start codon) when *SlDML2* was disrupted (Figure S10a). By contrast, an obvious DMR was identified in the first intron of *SlβCA3*, in which the 5mC levels of 16 representative cytosines were obviously increased in the *sldml2* mutant (Figure 5a). Bisulphite sequencing revealed that 11 of the 16 representative cytosines displayed higher 5mC levels in fruits of the *sldml2* mutants (*sldml2‐3* and *sldml2‐4*) than the wild‐type (Figure 5b), confirming the existence of DMR in the intronic region. The increase in 5mC levels of the intronic DMR was also observed in the *sldml2* mutants (*sldml2‐3* and *sldml2‐4*) after *B. cinerea* infection as revealed by McrBC‐PCR assay (Figure 5c). ChIP‐qPCR assay using the anti‐SlDML2 polyclonal antibody showed that SlDML2 protein can bind to the intronic DMR of *SlβCA3* gene (Figure 5d), indicating that *SlβCA3* is the direct target of SlDML2, which mediates 5mC level of *SlβCA3* in this region.

We then adopted a dual‐luciferase reporter system to determine whether *SlβCA3* is transcriptionally regulated by SlDML2‐mediated DNA demethylation (Han *et al*., 2016). The sequence fragment of *SlβCA3* promoter was cloned into the dual‐luciferase reporter vector pGreen II‐0800‐LUC to drive the expression of promoterless firefly luciferase (FLUC) reporter gene (Figure S10b). The transcription level of *FLUC* gene was relatively calculated by normalizing against the renilla luciferase (RLUC) reference gene driven by the CaMV 35S promoter. Quantitative RT‐PCR analysis showed that the *FLUC* transcription level was not increased under the expression of SlDML2‐HA fusion protein (Figure S10c,d), which is consistent with the observation that no DMR occurred in the *SlβCA3* promoter region under *SlDML2* disruption, suggesting that the *SlβCA3* promoter is indeed free from SlDML2‐mediated DNA demethylation.

We then asked whether SlDML2 regulates *SlβCA3* transcription through modulating 5mC level in the intronic DMR. The sequence of *SlβCA3* gene body containing all exons and introns was cloned into the dual‐luciferase reporter vector to replace the *FLUC* gene sequence for transcription activity assay under the driving of its native promoter. The *SlβCA3* transcription level increased significantly when SlDML2 was co‐expressed (Figure 5e), concomitant with a decline in 5mC level of the intronic DMR (Figure 5f). By contrast, SlDML2 co‐expression did not induce a significant increase in *SlβCA3* transcription when those representative cytosines in the intronic DMR were mutated to adenine or thymine (Figure 5g), indicating that *SlβCA3* could be transcriptionally regulated by SlDML2‐mediated DNA demethylation.

Taken together, these data suggest that *SlβCA3*, which could be transcriptionally activated by SlDML2 through active DNA demethylation, functions as a defence gene towards *B. cinerea* invasion, thus plays an important role in SlDML2‐mediated tomato resistance to *B. cinerea*.

### SlDML2‐mediated DNA demethylation regulates the expression of JA biosynthetic genes

Previous studies have shown that the phytohormone jasmonic acid (JA) plays an essential role in activating plant defence responses against fungal pathogens (Du *et al*., 2017). During *B. cinerea* infection, enhanced JA biosynthesis and signalling transduction induce a series of defence responses, which finally confers increased resistance of tomato fruits to *B. cinerea* (Huang *et al*., 2022; Martel *et al*., 2015; Reyes‐Diaz *et al*., 2016; Shu *et al*., 2021). To investigate whether JA pathway is involved in the elevated susceptibility of *sldml2* mutants to *B. cinerea* infection, the changes in transcription levels of JA biosynthetic genes were analysed based on our RNA‐seq data. JA biosynthesis is synergistically controlled by multiple gene families that encode distinct biosynthetic enzymes (Figure S11a). Heat map showed that some genes within those families exhibited lower transcription level in the *sldml2‐3* mutant than the wild‐type during *B. cinerea* invasion, including the *ω‐3 desaturase* 3 (*SlFAD3*) (Domínguez *et al*., 2010; Yu *et al*., 2009), the *lipoxygenase B/C/D* (*SlLOXB/C/D*) (Zhu‐Salzman *et al*., 2008), the *allene oxide synthase 1* (*SlAOS1*) (Sivasankar *et al*., 2000), the *allene oxide cyclase* (*SlAOC*) (Ziegler *et al*., 2000) and the *12‐oxophytodienoate reductase 3* (*SlOPR3*) (Strassner *et al*., 2002) (Figure S11a). The differential gene expression was further confirmed by quantitative RT‐PCR analysis (Figure S11b), indicating the involvement of SlDML2 in regulating the expression of JA biosynthetic genes.

**Figure 6:**
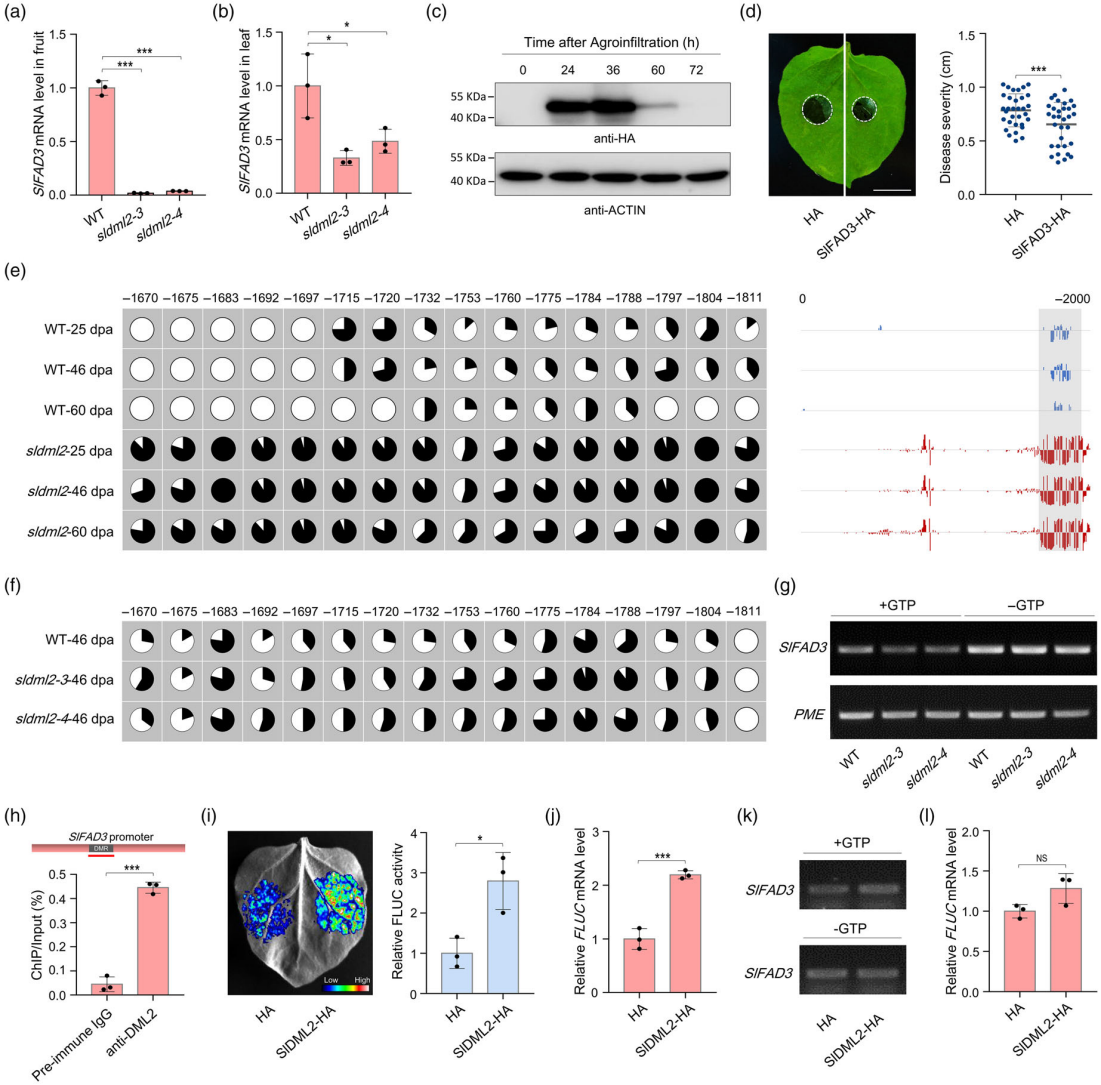
*SlFAD3* is transcriptionally regulated by SlDML2‐mediated DNA demethylation. (a) Transcription levels of the tomato ω‐3 fatty acid desaturase gene *SlFAD3* in fruits of the wild‐type (WT) and *sldml2* mutants after *B. cinerea* inoculation for 48 h. (b) Transcription levels of the *SlFAD3* gene in leaves of the WT and *sldml2* mutants after *B. cinerea* inoculation for 48 h. (c) Transient expression of SlFAD3‐HA protein in *N. benthamiana* leaves. Total protein was extracted at indicated time after agroinfiltration and then submitted to immunoblot with anti‐HA antibody. Equal loading was confirmed by using the tomato Actin as an internal control. (d) *B. cinerea* disease symptoms and lesion diameters on detached *N. benthamiana* leaves with or without the co‐expression of SlFAD3‐HA. Scale bar = 1 cm. (e) DNA methylation (5mC) levels of the *SlFAD3* promoter in fruits of the WT and *sldml2* mutant at indicated developmental stages. The 5mC level was analysed by using the published DNA methylome database (Lang *et al*., 2017). Each vertical bar represents a 5mC and the bar height indicates 5mC level. The differentially methylated region (DMR) in the *SlFAD3* promoter was indicated by a shadow box. 5mC levels of the representative cytosines in the DMR were shown with pie charts, and the numbers indicate the positions relative to the start codon. dpa, days post‐anthesis. (f) Bisulphite sequencing showing 5mC levels of the representative cytosines shown in (e) in fruits of the wild‐type and *sldml2* mutants (*sldml2‐3* and *sldml2‐4*) at 46 dpa. (g) 5mC level of the DMR of *SlFAD3* promoter in fruits of the WT and *sldml2* mutants after *B. cinerea* inoculation for 48 h. (h) ChIP‐qPCR assay showing that SlDML2 binds to the DMR in the promoter of *SlFAD3*. The wild‐type fruits at 42 dpa were subjected to the immunoprecipitation with the anti‐SlDML2 polyclonal antibody, and the pre‐immune IgG was used as a control. The promoter structure of *SlFAD3* is shown, and the red line indicates the region for PCR. (i) Relative firefly luciferase (FLUC) activity derived by the *SlFAD3* promoter in *N. benthamiana* leaves with or without the co‐expression of SlDML2‐HA. The representative image was shown. The FLUC activity was normalized against the renilla luciferase (RLUC) activity, followed by normalization against the control. (j) Transcription levels of the *FLUC* gene under the drive of *SlFAD3* promoter in *N. benthamiana* leaves with or without the co‐expression of SlDML2‐HA. The *RLUC* gene was used as an internal control. (k) 5mC level of the DMR of *SlFAD3* promoter in *N. benthamiana* leaves with or without the co‐expression of SlDML2‐HA. (l) Transcription levels of the *FLUC* gene under the drive of mutated *SlFAD3* promoter in *N. benthamiana* leaves with or without the co‐expression of SlDML2‐HA. The representative cytosines shown in (e) were mutated to adenine or thymine. Ns, no significance. In (g) and (k), total 5mC level was revealed by McrBC‐PCR assay. A total of 0.4 μg genomic DNA was digested by McrBC enzyme with GTP (+GTP), or without GTP (−GTP) as a negative control. An unmethylated region in the promoter of tomato *PME* gene was used as an internal control. In (a, b, d, h–j), asterisks indicate significant differences (*P < 0.05, ***P < 0.001; Student's *t* test).

Since the JA biosynthetic gene *SlFAD3*, which encodes one of the *ω‐*3 fatty acid desaturases that catalyse the conversion of linoleic acid (18:2) to linolenic acid (18:3) (Domínguez *et al*., 2010; Yu *et al*., 2009), exhibited a dramatically decreased expression when *SlDML2* was mutated (Figure S11b), we speculate that *SlFAD3* may be directly regulated by SlDML2‐mediated DNA demethylation and involved in the elevated susceptibility of *sldml2* mutants to *B. cinerea* infection. Compared to the wild‐type, the *SlFAD3* transcription level was obviously lower in fruits and leaves of the *sldml2‐3* and *sldml2‐4* mutants after *B. cinerea* infection (Figure 6a,b). Moreover, the expression of *SlFAD3* decreased obviously in fruits of the *sldml2* mutants under normal developmental process (Figure S7b), indicating that SlDML2 positively regulates *SlFAD3* expression.

We next investigated whether SlFAD3 functions in modulating *B. cinerea* disease resistance using the *N. benthamiana* expression system. SlFAD3 protein was transiently expressed in *N. benthamiana* leaves, and the *B. cinerea* was inoculated at the 36 h post‐agroinfiltration when the protein was obviously expressed (Figure 6c). Observation of the disease symptoms at the second day post‐inoculation revealed that the expression of SlFAD3 protein conferred the *N. benthamiana* leaves a significantly increased resistance to *B. cinerea* (Figure 6d), indicating that SlFAD3 harbours the capability of defensing *B. cinerea* invasion. We also analyzed the function of SlFAD3 in regulating resistance to *V. dahliae* (Figure S8) and *Pst* DC3000 (Figure S9b) but found that SlFAD3 was dispensable for resistance to both pathogens.

An obvious DMR was identified in the promoter region, rather than the gene body, of *SlFAD3* in the *sldml2* mutants, according to the published methylomes (Lang *et al*., 2017), in which the 5mC levels of 16 representative cytosines are obviously increased in the *sldml2* mutant compared to the wild‐type (Figure 6e). Bisulphite sequencing showed that 13 of the 16 representative cytosines exhibited higher 5mC levels in fruits of the *sldml2* mutants (*sldml2‐3* and *sldml2‐4*) than the wild‐type, confirming that DMR exists in the promoter region of *SlFAD3* (Figure 6f). The DMR also displayed higher 5mC level in fruits of the *sldml2‐3* and *sldml2‐4* mutants than those of the wild‐type during *B. cinerea* invasion (Figure 6g). ChIP‐qPCR assay revealed that SlDML2 protein can bind to the DMR in the promoter region of *SlFAD3* gene (Figure 6h), indicating that *SlFAD3* is the direct target of SlDML2, which mediates 5mC level of *SlFAD3* in the promoter.

To determine whether *SlFAD3* is transcriptionally regulated by SlDML2‐mediated DNA demethylation, the sequence fragment of *SlFAD3* promoter (~2000 bp upstream of the start codon) was cloned into the dual‐luciferase reporter vector to drive the expression of *FLUC* reporter gene. The relative FLUC activity and *FLUC* transcription level were significantly increased when SlDML2 was co‐expressed with the reporter vector (Figure 6i,j), concomitant with a decline in 5mC level in the DMR of *SlFAD3* promoter as determined by McrBC‐PCR assay (Figure 6k). Importantly, SlDML2 co‐expression only induced a slight increase in *FLUC* transcription (~1.3‐fold) when those representative cytosines in the DMR were mutated to adenine or thymine (Figure 6l). These results indicate that *SlFAD3* could be transcriptionally regulated by SlDML2‐mediated DNA demethylation.

Collectively, these data suggest that *SlFAD3*, which could be transcriptionally activated by SlDML2 through active DNA demethylation, functions as a defence gene towards *B. cinerea* invasion and, thus plays a critical role in SlDML2‐mediated tomato resistance to *B. cinerea*.

### *SlβCA3* and *SlFAD3* functions probably in the same genetic pathway with *SlDML2* to regulate tomato resistance to *B. cinerea*

**Figure 7:**
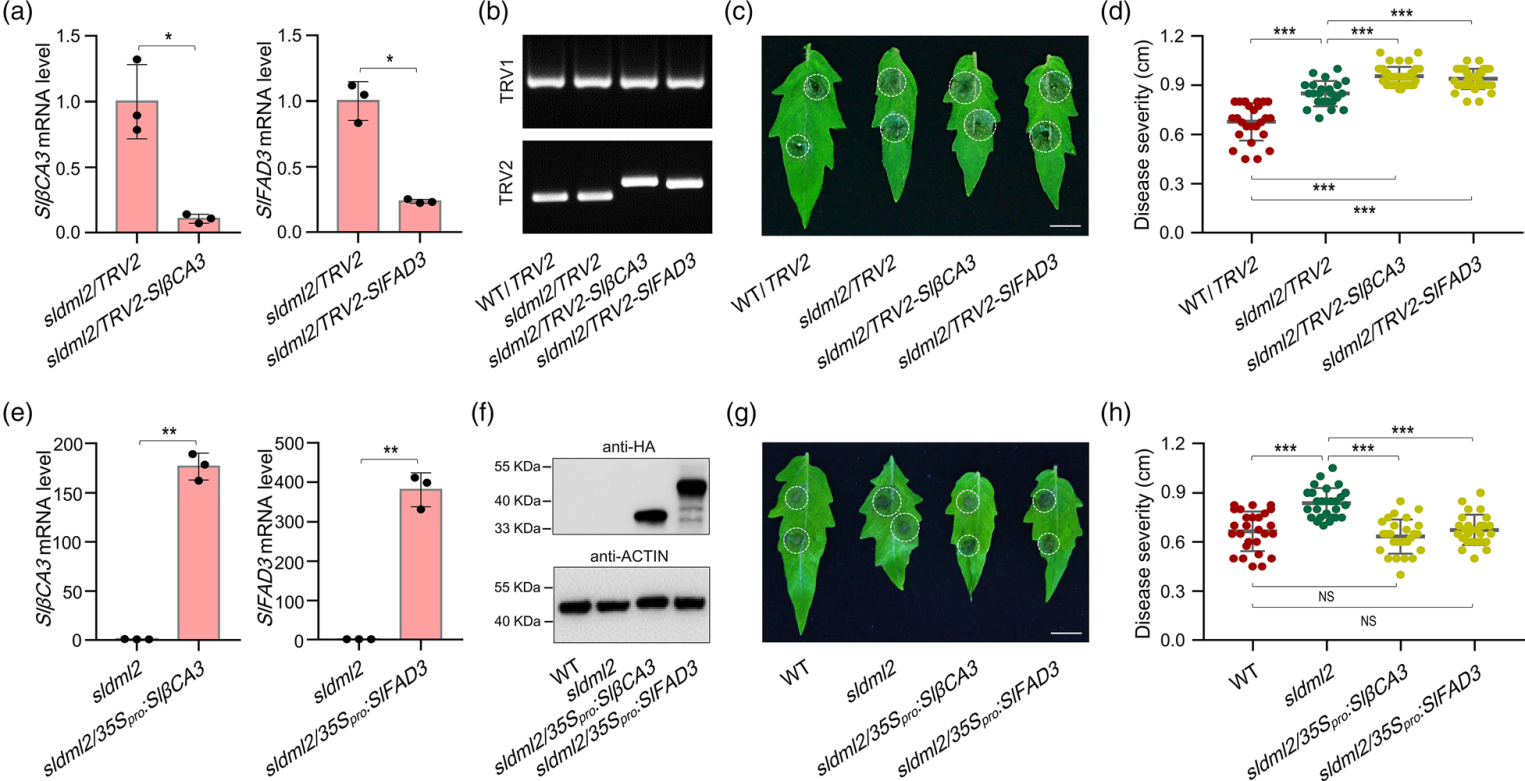
*SlDML2* functions in the same genetic pathway with *SlβCA3* and *SlFAD3* to regulate tomato resistance to *B. cinerea*. (a) Transcription levels of *SlβCA3* and *SlFAD3* in leaves of the *sldml2* mutant after virus‐induced gene silencing (VIGS), as determined by quantitative RT‐PCR. The *sldml2* mutant infiltrated with the empty vector pTRV2 was used as the control group. The tomato *Actin* gene was used as an internal control. (b) PCR amplification showing that the virus vectors pTRV1, pTRV2, pTRV2‐*SlβCA3*, and pTRV2‐*SlFAD3* were successfully expressed in leaves of the wild‐type (WT) or *sldml2* mutant. (c) Disease symptoms and (d) lesion diameters on leaves of the WT/*TRV2*, *sldml2/TRV2*, *sldml2/TRV2‐SlβCA3*, and *sldml2/TRV2‐SlFAD3* inoculated with *B. cinerea* for 40 h. (e) Transcription levels of *SlβCA3* and *SlFAD3* in leaves of the *sldml2* mutant after overexpression, as determined by quantitative RT‐PCR. The *sldml2* mutant infiltrated with the empty vector pCambia1302‐HA was used as the control groups. The tomato *Actin* gene was used as an internal control. (f) Western blot showing that SlβCA3‐HA and SlFAD3‐HA fusion proteins were successfully expressed in leaves of *sldml2/35S*_*pro*_ *:SlβCA3* And *sldml2/35S*_*pro*_ *:SlFAD3*. Total protein was extracted and submitted to immunoblot with anti‐HA antibody. Equal loading was confirmed by using the tomato Actin as an internal control. (g) Disease symptoms and (h) lesion diameters on leaves of the WT, *sldml2*, *sldml2/35S*_*pro*_ *:SlβCA3*, And *sldml2/35S*_*pro*_ *:SlFAD3* Inoculated with *B. cinerea* for 40 h. In (a, d, e, and h), asterisks indicate significant differences (*P < 0.05, **P < 0.01, ***P < 0.001; Student's *t* test). NS, no significance. In (c and g), scale bar = 1 cm.

To further verify the roles of *SlβCA3* and *SlFAD3* in SlDML2*‐*mediated tomato resistance to *B. cinerea*, we silenced *SlβCA3* or *SlFAD3* gene in the *sldml2* mutant (*sldml2‐3*) background by using virus‐induced gene silencing (VIGS), and then investigated whether this led to a decrease in resistance of the *sldml2* mutant to *B. cinerea*. Quantitative RT‐PCR analysis showed that the transcription levels of *SlβCA3* and *SlFAD3* in the VIGS materials (*sldml2/TRV2‐SlβCA3* and *sldml2/TRV2‐SlFAD3*) were decreased to almost 10% and 25% of the control group (*sldml2/TRV2*), respectively (Figure 7a). Meanwhile, RNA fragments transcribed from the virus vectors pTRV1, pTRV2, pTRV2‐*SlβCA3* and pTRV2‐*SlFAD3* were obviously detected in corresponding samples (Figure 7b). These results indicate that *SlβCA3* and *SlFAD3* gene were successfully silenced in the *sldml2* mutant. The leaves of the *sldml2* mutant displayed larger lesion diameters compared to the wild‐type, and the symptom severity was further increased in leaves of the *sldml2/TRV2‐SlβCA3* or *sldml2/TRV2‐SlFAD3* plants (Figure 7c,d), indicating an obviously additive effect on *B. cinerea* susceptibility induced by gene silencing of *SlβCA3* or *SlFAD3*. Furthermore, we also overexpressed *SlβCA3* or *SlFAD3 gene* in the *sldml2* mutant and analysed its resistance to *B. cinerea*. Quantitative RT‐PCR and immunoblot assays showed that the two genes were successfully overexpressed in leaves of the *sldml2* mutant (Figure 7e,f). Compared to the *sldml2* mutant, the two overexpression materials (*sldml2/35S*_*pro*_ *:SlβCA3* and *sldml2/35S*_*pro*_ *:SlFAD3*) exhibited significantly smaller lesion diameters, which were equally to those of the wild‐type, indicating that overexpression of *SlβCA3* or *SlFAD3* in the *sldml2* mutant can recover the resistance to *B. cinerea* (Figure 7g,h). Taken together, these data suggest that *SlβCA3* and *SlFAD3* may regulate tomato resistance to *B. cinerea* in the same genetic pathway with *SlDML2*.

### *B. cinerea* invasion causes a ripening delay in the pericarp tissues surrounding the infection region

**Figure 8:**
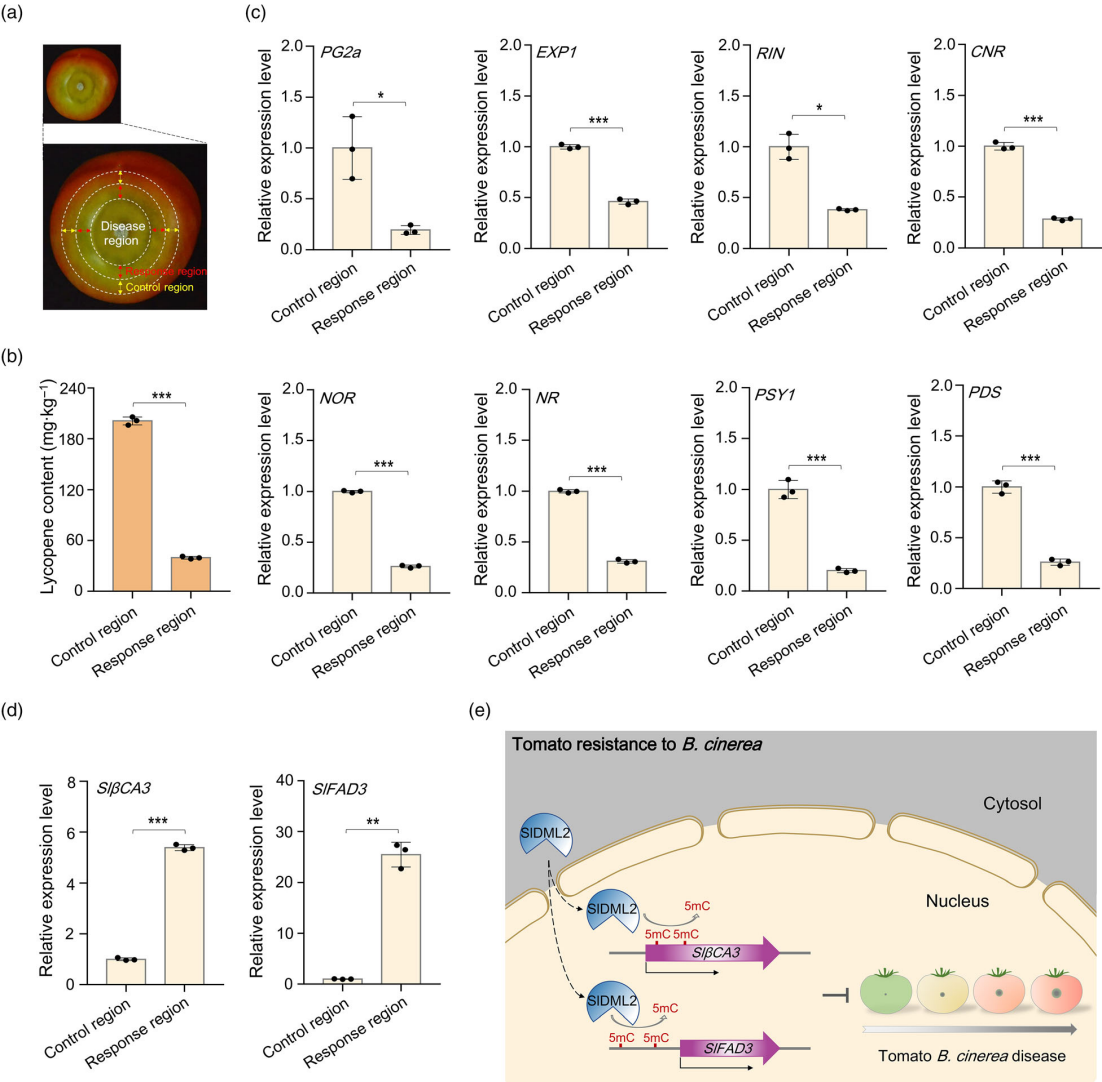
*B. cinerea* invasion causes a delay in ripening of pericarp tissues around the disease region. (a) Representative photograph of wild‐type tomato fruits after *B. cinerea* inoculation for 4 days. The pericarp tissue with a 5‐mm thickness surrounding disease regions was collected as “response region”, and the pericarp tissue with a 5‐mm thickness around the response region was collected as “control region”. (b) Lycopene content of the response region and control region. (c) Transcription levels of ripening‐related genes in the response region and control region as determined by quantitative RT‐PCR analysis. The tomato *SlUBI3* gene was used as an internal control. *PG2a*, *polygalacturonase 2a*; *EXP1*, *expansion 1*; *RIN*, *ripening inhibitor*; *CNR*, *colourless nonripening*; *NOR*, *nonripening*; *NR*, *never ripe*; *PSY1*, *phytoene synthase 1*; *PDS*, *phytoene desaturase*. (d) Transcription levels of *SlβCA3* and *SlFAD3* in the response region and control region as determined by quantitative RT‐PCR analysis. The tomato *SlUBI3* gene was used as an internal control. In (b–d), asterisks indicate significant differences (*P < 0.05, **P < 0.01, ***P < 0.001; Student's *t* test). (e) Model for SlDML2‐mediated regulation of tomato resistance to *B. cinerea*. SlDML2 transcriptionally activates the expression of defence‐related genes *SlβCA3* and *SlFAD3* through active DNA demethylation in the gene body and promoter region, respectively, thus positively regulating tomato fruit resistance to *B. cinerea*.

At the fourth day after *B. cinerea* infection, we observed that the pericarp of wild‐type tomato fruits primarily exhibited a homogeneous orange colour, likely due to continual postharvest ripening. However, the pericarp tissue around *B. cinerea* disease region displayed green colour of different degree (Figure S12a), indicating a regional ripening delay caused by *B. cinerea* infection. We defined the pericarp tissue with a 5‐mm thickness surrounding disease regions as the response region of *B. cinerea* infection, and the pericarp tissue with a 5‐mm thickness around the response region as the control region (Figure 8a). Compared to the control region, the response region contained significantly decreased lycopene content (Figure 8b). Furthermore, transcription levels of several known ripening genes were obviously lower in the response region than in the control region (Figure 8c), and this decrease in gene expression was observed even in fruits that did not exhibit a macroscopic ripening delay in the response region (Figure S12b). These results suggest that *B. cinerea* invasion causes a ripening delay in the pericarp tissues surrounding the infection region.

Considering that ripe tomato fruits are more susceptible to *B. cinerea* than unripe green fruits (Cantu *et al*., 2009; Prusky *et al*., 2013; Silva *et al*., 2023), we referred to the delayed ripening under *B. cinerea* invasion as a self‐protection mechanism of tomato fruits. It is possible that tomato fruit pericarp around disease region tend to abandon normal ripening process for obtaining increased defence capability in face with *B. cinerea* infection, thus achieving the balance between ripening and disease resistance. The ripening genes exhibiting decreased transcription level in the response region appeared to be the targets of SlDML2 (Figure 8c) (Lang *et al*., 2017). By contrast, the defence genes *SlβCA3* and *SlFAD3* exhibited significantly increased expression in the response region compared with the control region (Figure 8d). Accordingly, it is possible that SlDML2 preferentially activates the expression of defence genes, rather than those ripening genes, to confer tomato fruits increased resistance as far as possible and plays an essential role in mediating the trade‐off between fruit ripening and disease resistance during the pathogenic process of *B. cinerea*. Notably, *B. cinerea* invasion induced a decrease in *SlDML2* mRNA level in the response region compared with the control region (Figure S13), suggesting that the delay in ripening of the pericarp tissue surrounding *B. cinerea* infection and the decrease in expression of ripening genes targeted by SlDML2 might be caused by the decreased expression of *SlDML2* in this region.

## Discussion

### The DNA demethylase gene *SlDML2* functions as a multifunctional gene

DEMETER/DEMETER‐like DNA demethylases contain the conserved domain that non‐specifically recognizes DNA sequence, which is generally thought to confer the DNA demethylases broad targets and diversified functions (Mok *et al*., 2010). Indeed, Arabidopsis DEMETER DNA demethylase gene *AtDME* has been demonstrated to modulate many aspects of sporophytic growth and development, including seed germination, root hair growth and de novo shoot formation (Kim *et al*., 2021). Moreover, the Arabidopsis DNA demethylase gene *AtROS1*, the ortholog of *AtDME*, participates in regulating the development of stomatal lineage cells, seed dormancy and the resistance responses to *Hyaloperonospora arabidopsidis* through active DNA demethylation (Yamamuro *et al*., 2014). However, *SlDML2*, the tomato homologue of *AtROS1*, has only been characterized as a pivotal ripening gene so far (Gao *et al*., 2022; Lang *et al*., 2017; Liu *et al*., 2015; Zhou *et al*., 2019). In this study, we generated the *sldml2* homozygous mutants using CRIPSR/Cas9 gene‐editing system and found that *sldml2* mutants exhibited obvious development defects in seeds, leaves and flowers, in addition to the delay in fruit ripening (Figure 1). We also reported here that SlDML2 positively regulates tomato resistance to the fungal pathogens *B. cinerea* and *V. dahliae* (Figure 2). Thus, our study extends the molecular function of *SlDML2* to plant organ development and disease resistance and uncovers the *SlDML2* as a multifunctional gene.

### SlDML2‐mediated DNA demethylation facilitates the expression of defence‐related genes

Previous studies have suggested that SlDML2‐mediated DNA demethylation promotes the expression of hundreds of ripening genes, and thus facilitates tomato fruit ripening (Gao *et al*., 2022; Lang *et al*., 2017; Li *et al*., 2020; Zhou *et al*., 2019). In this study, we found that *SlDML2* mutation weakened tomato resistance to *B. cinerea* and decreased the expression of plenty of defence‐related genes during *B. cinerea* invasion (Figure 2; Table S3). Accordingly, it is possible that SlDML2‐mediated DNA demethylation facilitates tomato resistance to *B. cinerea* by activating the transcription of plenty of defence‐related genes, similar to the ripening regulation that involves numerous ripening genes. To decipher the molecular basis underlying SlDML2‐mediated disease resistance, we focused on the β‐type carbonic anhydrase gene *SlβCA3* for the reason that it has been identified as an important defence gene against the virulent pathogen *Pst* DC3000 (Hu *et al*., 2021), and the JA biosynthetic gene *SlFAD3* because of the functional importance of JA pathway in defensing *B. cinerea* infection (Huang *et al*., 2022; Reyes‐Diaz *et al*., 2016; Shu *et al*., 2021). Although the two defence genes were revealed to be transcriptionally regulated by SlDML2 through active DNA demethylation and play an important role in the defence response against *B. cinerea* (Figures 4, 5, 6), it is possible that SlDML2 also targets other defence‐related genes, such as those in JA biosynthesis and signalling transduction pathway, to modulate tomato resistance to *B. cinerea*.

DNA methylation occurs in distinct sequences of genome, including promoters, gene bodies, transposable elements and intergenic regions (Tang *et al*., 2016; Zhang *et al*., 2018). Although SlDML2 was previously demonstrated to preferentially target promoters, transposable elements and intergenic regions, like AtROS1 in Arabidopsis (Tang *et al*., 2016), there still exist approximately 10% of SlDML2 targets located in gene body regions (Lang *et al*., 2017). In contrast to the role of promoter methylation in gene expression, the role of SlDML2‐targeted methylation of gene bodies in gene regulation is less characterized. In Arabidopsis, gene body methylation in the CG context exhibited a positive correlation with gene expression (Schmitz *et al*., 2013; Zilberman *et al*., 2007), while those in the CHG and CHH contexts tend to negatively regulate gene expression (You *et al*., 2012). The function in gene expression regulation mediated by gene body methylation was also revealed in other plant species, such as rice (Wang *et al*., 2017c), tomato (González *et al*., 2011) and common bean (Richard *et al*., 2018). In this study, we found that SlDML2‐mediated DNA demethylation in the first intron of *SlβCA3* gene body facilitated its transcription (Figure 5), indicating a positive regulation of SlDML2 in gene expression by 5mC removal of the intronic regions. However, the underlying molecular basis is currently elusive. It is possible that SlDML2‐mediated intronic DNA demethylation regulates gene expression by associating with multiple events. For example, DNA demethylation in intron‐exon boundaries may impact alternative splicing, thereby modulating gene expression (Regulski *et al*., 2013).

### SlDML2 may be an essential factor in modulating the trade‐off between fruit ripening and disease resistance

Fruit ripening and disease resistance are two tightly interconnected processes, as fleshy fruit exhibits increased susceptibility to *B. cinerea* with the ripening (Cantu *et al*., 2009), and *B. cinerea* infection induces the early‐ripe of unripe fruits (Silva *et al*., 2023). It is further supported by the cases that some ripening genes participate in regulating tomato resistance to *B. cinerea*, such as the vacuolar protease gene *SlVPE3* (Wang *et al*., 2017b), the cell wall‐modifying gene *SlPG* and *SlEXP1* (Cantu *et al*., 2008; Perini *et al*., 2017), and the transcription factor gene *SlNOR* (Cantu *et al*., 2009). In this study, the involvement of the pivotal ripening gene *SlDML2* in tomato resistance to *B. cinerea* invasion was revealed (Figure 2), which indicates the functional importance of *SlDML2* in simultaneously regulating fruit ripening and disease resistance.

*B. cinerea* infection is generally thought to accelerate tomato fruit ripening, partially through inducing ethylene synthesis and promoting respiration rate (Cristescu *et al*., 2002; Silva *et al*., 2023). This ripening‐promoting effect mainly refers to the whole unripe fruit. Indeed, we found that *B. cinerea* infection induced the pericarp tissues surrounding disease regions a delay in ripening (Figure 8a–c). Tomato fruits tend to abandon normal ripening process for obtaining enhanced defence capability when threated by continuous *B. cinerea* infection, known as the trade‐off between fruit ripening and disease resistance, as tomato fruits commonly exhibits increased susceptibility to *B. cinerea* with the progress of maturity. Therefore, the regional ripening inhibitory we observed could be an important self‐protection mechanism for tomato fruits under *B. cinerea* invasion.

A previous comparative transcriptomic analysis indicates that *SlDML2* mutation decreased the expression of hundreds of ripening‐related genes during tomato fruit ripening, including those regulating pigment synthesis, flavonoid synthesis and fruit softening (Lang *et al*., 2017). As a comparison, our comparative transcriptomic analysis between tomato fruits of the wild‐type and *sldml2* mutants during the pathogenic process of *B. cinerea* revealed that the down‐regulated genes in the *sldml2* mutant fruit were most significantly enriched in defence response to biotic and abiotic stresses, including ‘defense response to fungus’, ‘response to chitin’, ‘response to wounding’, ‘response to oxidative stress’ and ‘cell wall organization’ (Table S3). It implicates that SlDML2 may preferentially target defence‐related genes to promote their expressions during *B. cinerea* infection, rather than those ripening genes. Consistent with this inference, several *SlDML2*‐targeted ripening genes exhibited decreased transcription levels in the pericarp tissue surrounding *B. cinerea* infection compared with the control (Figure 8c), whereas the expression of defence‐related genes *SlβCA3* and *SlFAD3* increased obviously (Figure 8d). Thus, we speculate that SlDML2 may participate in modulating the trade‐off between fruit ripening and disease resistance during *B. cinerea* invasion, although the underlying mechanisms remain to be further investigated.

In summary, our data revealed SlDML2‐mediated regulatory mechanism in tomato resistance against the fungal pathogen *B. cinerea*, wherein two defence‐related genes *SlβCA3* and *SlFAD3* were focused and proved to play important roles (Figure 8e). In addition, we reported here that SlDML2 participates in modulating multiple developmental processes besides fruit ripening. Thus, we defined the DNA demethylase gene *SlDML2* as a multifunctional gene, rather than merely a ripening gene. This study provided us new perspectives concerning the physiological function of *SlDML2* and facilitated the potential application of this essential regulatory gene in the future improvement of tomato.

## Materials and methods

### Plant materials and culture conditions

Seeds of wild‐type tomato (*Solanum lycopersicum* cv. Ailsa Craig) were obtained from Tomato genetics resource center (TGRC, https://tgrc.ucdavis.edu/policy.aspx). The *sldml2* mutants in Ailsa Craig background were produced by CRISPR/Cas9 gene‐editing as described below. Tomato seedlings were cultured in a greenhouse with 25 °C, 60%–80% relative humidity and a 16/8 h light/dark photoperiod. Tomato flowers were tagged at anthesis to determine the ripening stages of fruits. For the ripening phenotype observation, fruits of the wild‐type and *sldml2* mutants were harvested at 39, 42, 47 and 52 days post‐anthesis (dpa), when the wild‐type fruits reach to mature green (MG), breaker (Br), orange ripe (OR) and red ripe (RR) stages, respectively.

### CRISPR/Cas9‐mediated gene editing

CRISPR/Cas9‐mediated gene editing was performed as the described (Ma *et al*., 2015). Briefly, three specific targets were designed using CRISPR‐P 2.0 (http://crispr.hzau.edu.cn/CRISPR2/) and then inserted into sgRNA expression cassettes containing the AtU3b, AtU6‐1 and AtU6‐29 promoters, respectively. The constructed sgRNA expression cassettes were introduced into the pYLCRISPR/Cas9Pubi‐H binary vector with the Golden Gate method (Engler and Marillonnet, 2014). The resulting vector was transformed into *Agrobacterium tumefaciens* strain GV3101. The Agrobacterium was cultured in Luria‐Bertani (LB) liquid medium containing 50 μg mL^−1^ kanamycin, 50 μg mL^−1^ gentamycin and 50 μg ml^−1^ rifampicin at 28 °C for 24 h and then utilized to infect the cotyledons of wild‐type tomato at a final OD_600_ of 0.5. Following the standard tissue culture described by Fillatti *et al*. (1987), transgenic seedlings were obtained, and the genotypes were analysed by PCR detection with primers flanking the targets. The homozygous seedlings were remained and cultured for subsequent studies. Sequences of primers used for PCR amplification are listed in Table S5.

### *B. cinerea* culture condition and disease symptom assay

*B. cinerea* strain B05.10 was cultured on potato dextrose agar (PDA) medium at 22 °C and a 16/8 h light/dark photoperiod for 10 days. Then, the *B. cinerea* was collected in 1/2 potato dextrose broth (PDB) medium, followed by vortex for 30 s. The spores were separated from mycelium by filtration through two layers of sterile gauze, diluted in 1/2 PDB medium to a final concentration of 5 × 10^5^ spores per millilitre, and finally used for inoculation. For the disease symptom assay in tomato, 5 μL spore suspension of *B. cinerea* was pipetted onto pre‐wound fruits at 39 and 90 dpa or 4‐week‐old detached leaves. For the disease symptom assay in *N. benthamiana* leaves, 5 μL spore suspension of *B. cinerea* was pipetted onto detached *N. benthamiana* leaves. The inoculated fruits and leaves were placed into enclosed plastic crates for maintaining high‐relative humidity, and then incubated in a growth room with 23 °C and a 16/8 h light/dark photoperiod. Disease lesion diameters were measured with a ruler and analysed by the crossing method (Li *et al*., 2022). The experiment was performed with three independent biological replicates.

### RNA‐seq and data analysis

RNA‐seq was performed in *B. cinerea*‐infected fruits of the wild‐type and *sldml2‐3* mutant. Total RNAs were extracted from the 5 mm‐width pericarp tissue surrounding disease regions with hot‐phenol according to the method of Moore *et al*. (2005). RNA quantity and purity were analysed by Bioanalyzer 2100 (Agilent, G2939A) and RNA 6000 Nano LabChip kit (Agilent, 5067‐1511), respectively. About 2 μg high‐quality RNA samples with an RNA integrity (RIN) number >7.0 were employed to library construction using NEBNext ultra RNA library prepare kit (NEB, E7530), according to the standard protocols. The constructed libraries were subsequently sequenced on an Illumina Novaseq™ 6000 sequencer with a paired‐end read length of 150 bp. The sequencing contains three independent biological replicates, and each RNA sample was extracted from at least six inoculated tomato fruits to avoid individual difference.

Raw sequencing reads containing adapters or low‐quality bases were filtered by Cutadapt (Version 1.9) (Martin, 2011) to obtain clean reads. The clean reads were aligned to the tomato build_SL3.0 reference genome (ftp://ftp.solgenomics.net/tomato_genome/) using HISAT2 package (Version 2.2.1) (Kim *et al*., 2019). The uniquely mapped reads were assembled by StringTie (Version 2.1.6) with default parameters (Pertea *et al*., 2016). Then, the gene expression value FPKM (fragment per kilobase of transcript per million mapped reads) was estimated and calculated by StringTie and ballgown (Pertea *et al*., 2016). Comparative transcriptomic analysis was performed by DESeq2 (Love *et al*., 2014), and genes with an adjusted *P* value <0.05 and FPKM fold change ≥2 were considered as differentially expressed genes. GO enrichment was performed on Gene Ontology Consortium (http://www.geneontology.org/) and only statistically significant results with a Bonferroni‐corrected *P* value <0.05 were remained. KEGG (Kyoto Encyclopedia of Genes and Genomes) enrichment was carried out on the KEGG Database (https://www.kegg.jp/kegg/) with default parameters.

### Quantitative RT‐PCR analysis

Total RNAs (2 μg) were reverse transcribed into cDNAs using the HiScript® III RT SuperMix for qPCR kit (Vazyme, R323‐01). The synthetic cDNAs were then employed as templates for PCR amplification with the ChemQ universal SYBR qPCR master mix (Vazyme, Q711‐02‐AA) and a StepOne plus real‐time PCR system (Applied Biosystems). The PCR program (20 μL) was as follows: 95 °C for 10 min, followed by 40 cycles of 95 °C for 15 s and 60 °C for 30 s. Transcription level was relatively calculated by normalizing to reference genes using the cycle threshold (CT) 2^(−ΔCT)^ method (Schmittgen and Livak, 2008). Sequences of primers used for PCR amplification are listed in Table S5.

Details of the other methods are provided in Methods S1–S11.

## Funding

This work was supported by the National Natural Science Foundation of China (31925035 and 32202557), the China Postdocral Science Foundation (2022M723371) and the Shandong Provincial Key Research and Development Program (2022TZXD0023).

## Conflict of interest statement

The authors declare no conflicts of interest.

## Author contributions

G.Q. and L.Z. conceived and designed the experiments. S.T. and W.W. provided critical discussions. L.Z., G.G. and X.L. performed the experiments and analysed the data. L.Z. and G.Q. wrote the manuscript.

## Supporting information


**Figure S1** Genotypes and predicted peptides of the *SlDML2* locus in *sldml2* mutants generated by CRISPR/Cas9‐mediated gene editing.
**Figure S2** Fruits of *sldml2* mutants can turn red at the final ripe stage.
**Figure S3** *SlDML2* mutation does not cause significant difference in seed appearance.
**Figure S4** *SlDML2* gene expression in various tomato organs as determined by quantitative RT‐PCR.
**Figure S5** *SlDML2* mutation does not cause significant difference in length of seedling stem.
**Figure S6** Pearson correlation coefficients between RNA‐seq samples.
**Figure S7** *SlDML2* mutation impairs the gene expression of *SlβCA3* and *SlFAD3*.
**Figure S8** *SlβCA3* and *SlFAD3* show no effect on tomato resistance to *V. dahliae*.
**Figure S9** Roles of *SlβCA3* and *SlFAD3* in regulating resistance to *Pst* DC3000.
**Figure S10** *SlDML2* mutation does not cause differential 5mC modification in the *SlβCA3* promoter.
**Figure S11** *SlDML2* mutation disturbs the expression of JA biosynthetic genes.
**Figure S12** *B. cinerea* invasion causes a delay in ripening of tomato pericarp tissues around the disease region.
**Figure S13** Influence of *B. cinerea* invasion on *SlDML2* gene expression.
**Method S1** *V. dahliae* culture condition and disease symptom assay.
**Method S2** *Pst DC3000* culture condition and disease symptom assay.
**Method S3** 5mC assay by bisulphite sequencing.
**Method S4** ChIP‐qPCR assay.
**Method S5** McrBC‐PCR assay.
**Method S6** Protein extraction and western blot.
**Method S7** VIGS.
**Method S8** Transient overexpression.
**Method S9** Lycopene content measurement.
**Method S10** Transcription activity assay.
**Method S11** Data analysis.
**Table S1** Up‐regulated genes in the fruit of *sldml2‐3* mutant during the pathogenic process of *B. cinerea* compared with the wild‐type.
**Table S2** Down‐regulated genes in the fruit of *sldml2‐3* mutant during the pathogenic process of *B. cinerea* compared with the wild‐type.
**Table S3** Gene ontology (GO) enrichment analysis of differentially expressed genes in the fruit of *sldml2‐3* mutant during the pathogenic process of *B. cinerea* compared with the wild‐type.
**Table S4** Down‐regulated genes in the fruit of *sldml2‐3* mutant that was previously reported to harbour hypermethylated DMRs when the *SlDML2* was disrupted.
**Table S5** A summary of primer informations.

## Data Availability

The raw sequencing data from the RNA‐seq assay have been deposited in the Gene Expression Omnibus database under the accession number GSE223774. All the other support data are included in the article or the supplemental files.
