# Supplementary material for: The pivotal ripening gene SlDML2 participates in regulating disease resistance in tomato
Source: Plant Biotechnol J. 2023 Jul 19;21(11):2291–306. doi: 10.1111/pbi.14130 (PMC10579708; doi:10.1111/pbi.14130)
Supplement: Supplementary file 1 — Figure S1 Genotypes and predicted peptides of the SlDML2 locus in sldml2 mutants generated by CRISPR/Cas9‐mediated gene editing. Figure S2 Fruits of sldml2 mutants can turn red at the final ripe stage. Figure S3 SlDML2 mutation does not cause significant difference in seed appearance. Figure S4 SlDML2 gene expression in various tomato organs as determined by quantitative RT‐PCR. Figure S5 SlDML2 mutation does not cause significant difference in length of seedling stem. Figure S6 Pearson correlation coefficients between RNA‐seq samples. Figure S7 SlDML2 mutation impairs the gene expression of SlβCA3 and SlFAD3. Figure S8 SlβCA3 and SlFAD3 show no effect on tomato resistance to V. dahliae. Figure S9 Roles of SlβCA3 and SlFAD3 in regulating resistance to Pst DC3000. Figure S10 SlDML2 mutation does not cause differential 5mC modification in the SlβCA3 promoter. Figure S11 SlDML2 mutation disturbs the expression of JA biosynthetic genes. Figure S12 B. cinerea invasion causes a delay in ripening of tomato pericarp tissues around the disease region. Figure S13 Influence of B. cinerea invasion on SlDML2 gene expression. Method S1 V. dahliae culture condition and disease symptom assay. Method S2 Pst DC3000 culture condition and disease symptom assay. Method S3 5mC assay by bisulphite sequencing. Method S4 ChIP‐qPCR assay. Method S5 McrBC‐PCR assay. Method S6 Protein extraction and western blot. Method S7 VIGS. Method S8 Transient overexpression. Method S9 Lycopene content measurement. Method S10 Transcription activity assay. Method S11 Data analysis. Table S1 Up‐regulated genes in the fruit of sldml2‐3 mutant during the pathogenic process of B. cinerea compared with the wild‐type. Table S2 Down‐regulated genes in the fruit of sldml2‐3 mutant during the pathogenic process of B. cinerea compared with the wild‐type. Table S3 Gene ontology (GO) enrichment analysis of differentially expressed genes in the fruit of sldml2‐3 mutant during the pathogenic process of B. cinerea compar [file PBI-21-2291-s001.zip › pbi14130-sup-0001-Figures S1-S13.pdf]

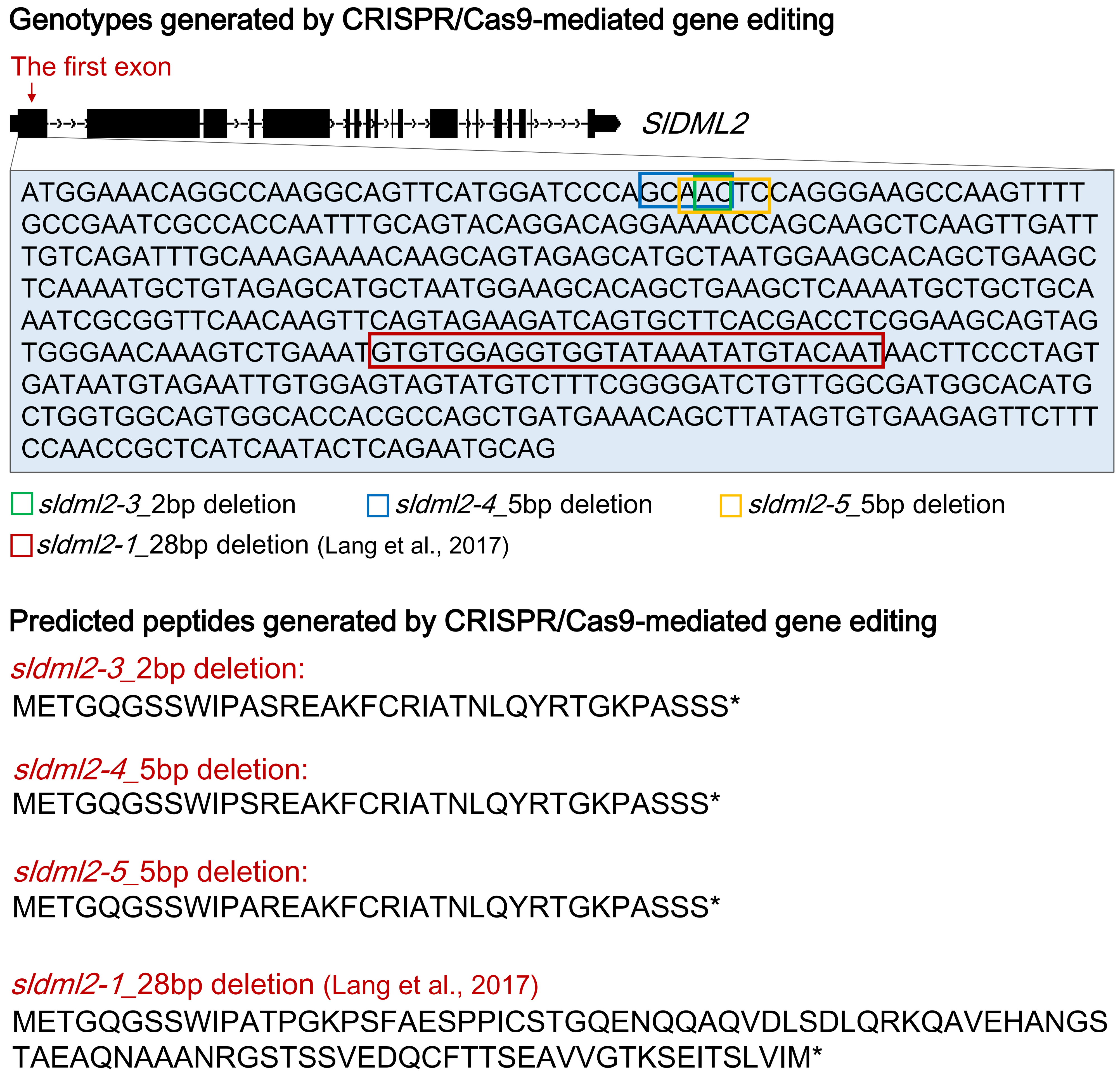

Figure S1 Genotypes and predicted peptides of SlDML2 in *sldml2* mutants generated by CRISPR/Cas9-mediated gene editing. Compared to the previously reported *sldml2-1* mutant produced by Lang et al. 2017, the *sldml2-3*, *sldml2-4*, and *sldml2-5* mutants were edited at the position closer to start codon in the first exon, and were predicted to produce shorter peptides.

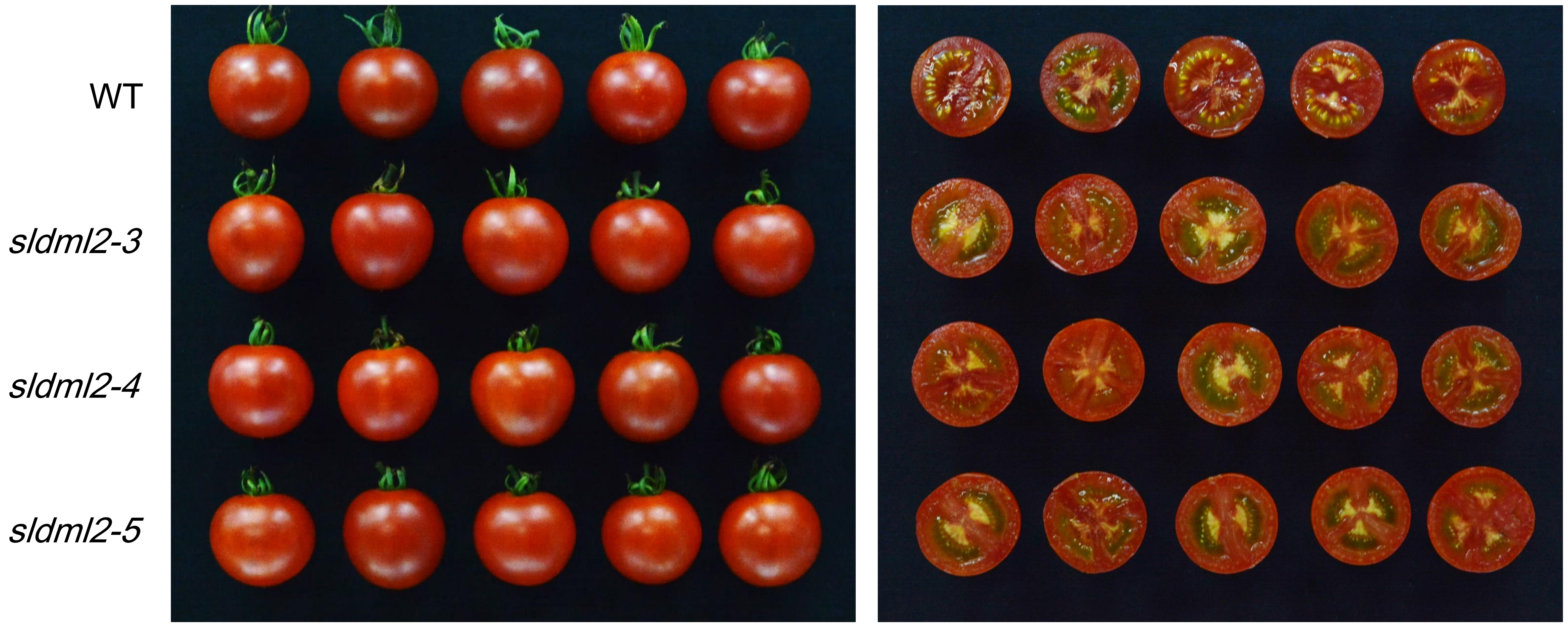

Figure S2 Fruits of *sldml2* mutants can turn red at the final ripe stage. Representative photographs of the wild-type (WT), *sldml2-3*, *sldml2-4*, and *sldml2-5* fruits at 90 days post-anthesis are shown.

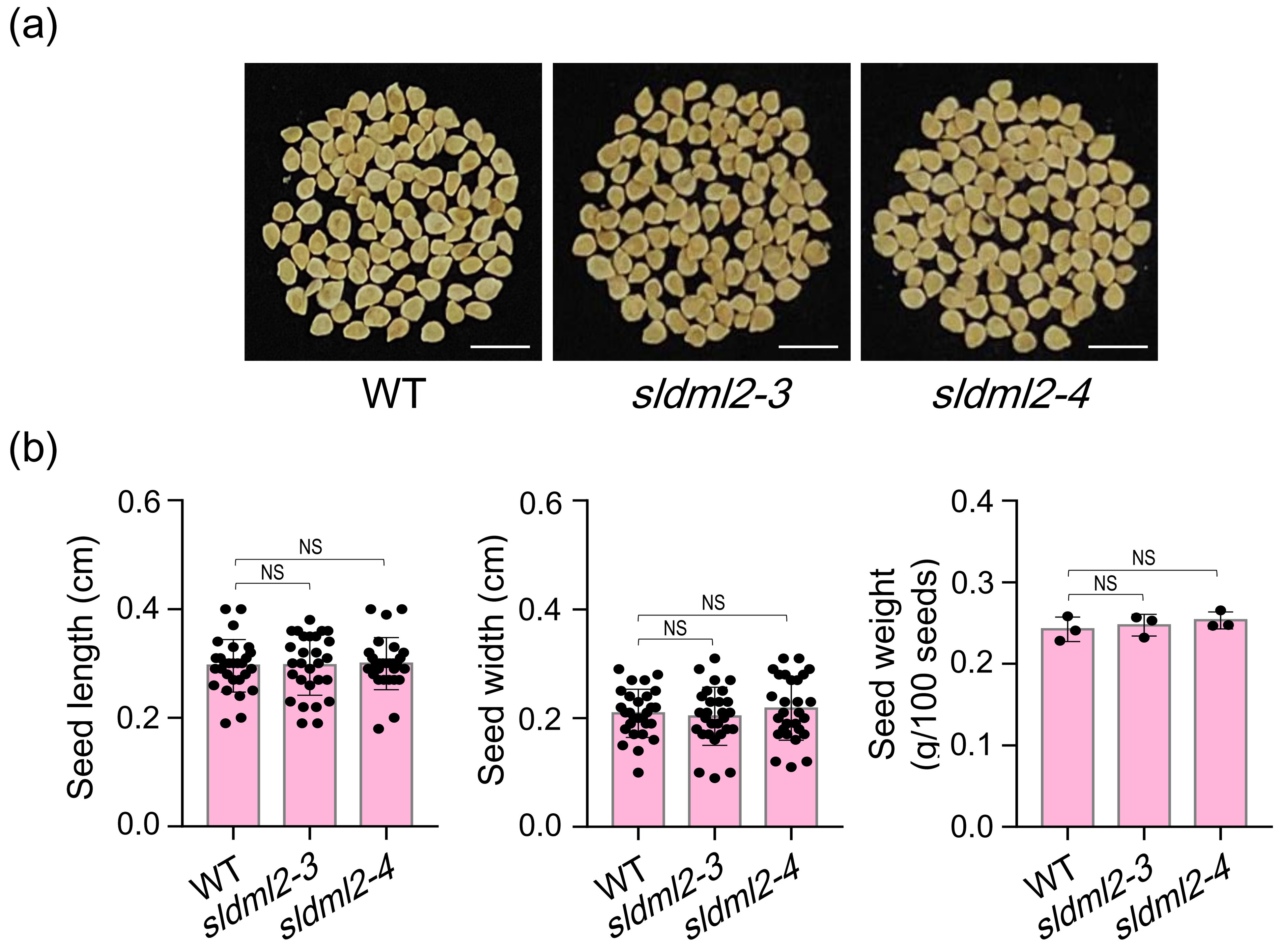

Figure S3 *SlDML2* mutation does not cause significant difference in seed appearance. (a) Representative photographs of the wild-type (WT), *sldml2-3*, and *sldml2-4* seeds. Scale bar = 1 cm. (b) Length, width, and weight of the WT, *sldml2-3*, and *sldml2-4* seeds. NS, no significance.

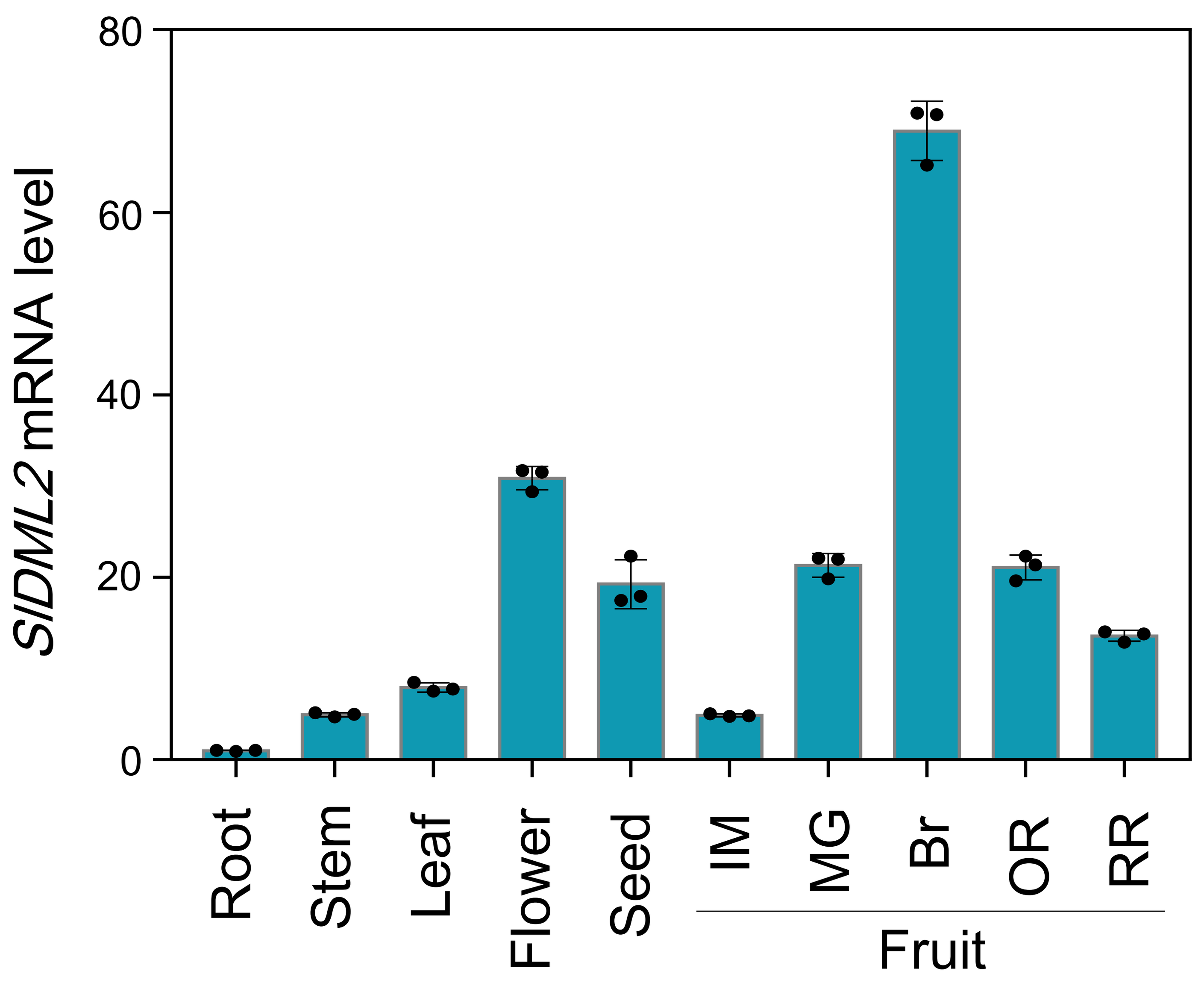

Figure S4 *SlDML2* gene expression in various tomato organs as determined by quantitative RT-PCR. The tomato *SlUBI3* gene was used as an internal control. IM, immature; MG, mature green; Br, breaker; OR, orange ripe; RR, red ripe.

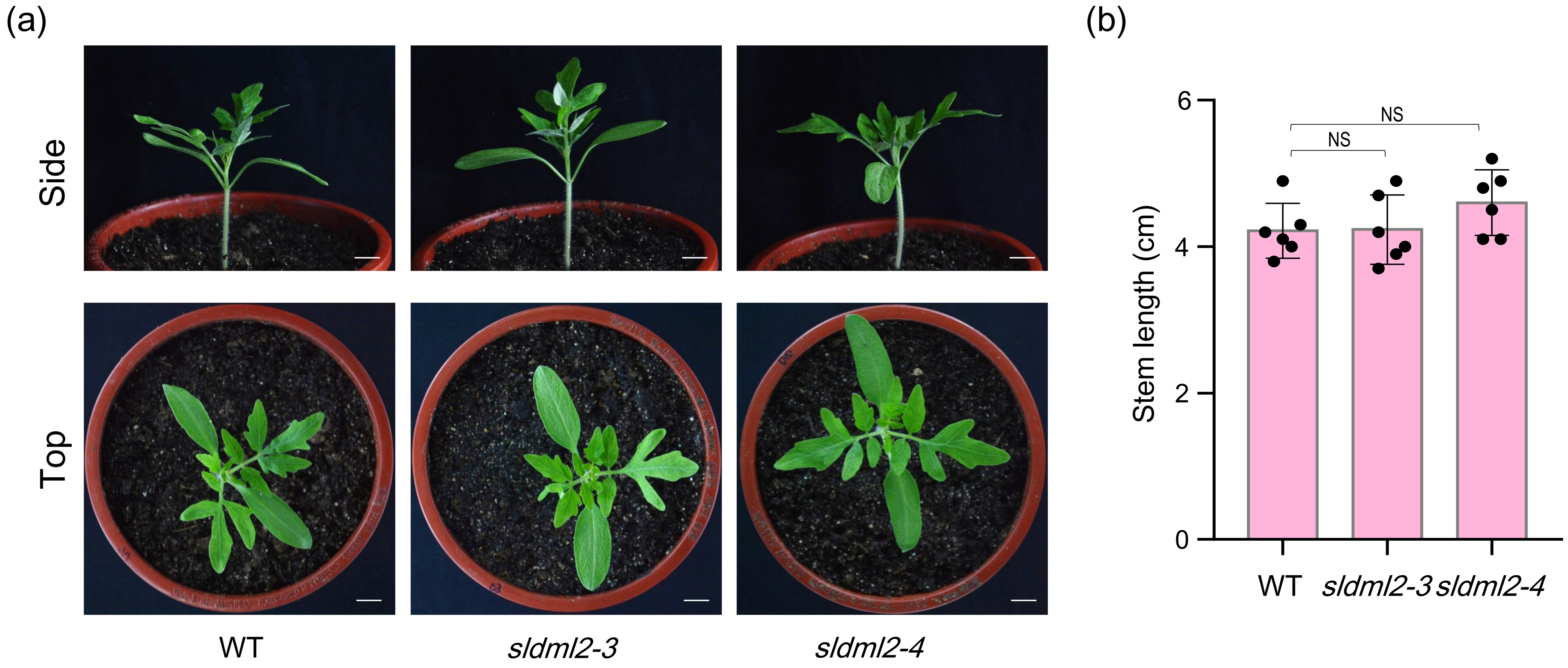

Figure S5 *SlDML2* mutation does not cause significant difference in length of seedling stem. (a) Representative photographs and (b) stem lengths of three-week-old wild-type (WT), *sldml2-3*, and *sldml2-4* seedlings. Scale bar = 1 cm. NS, no significance.

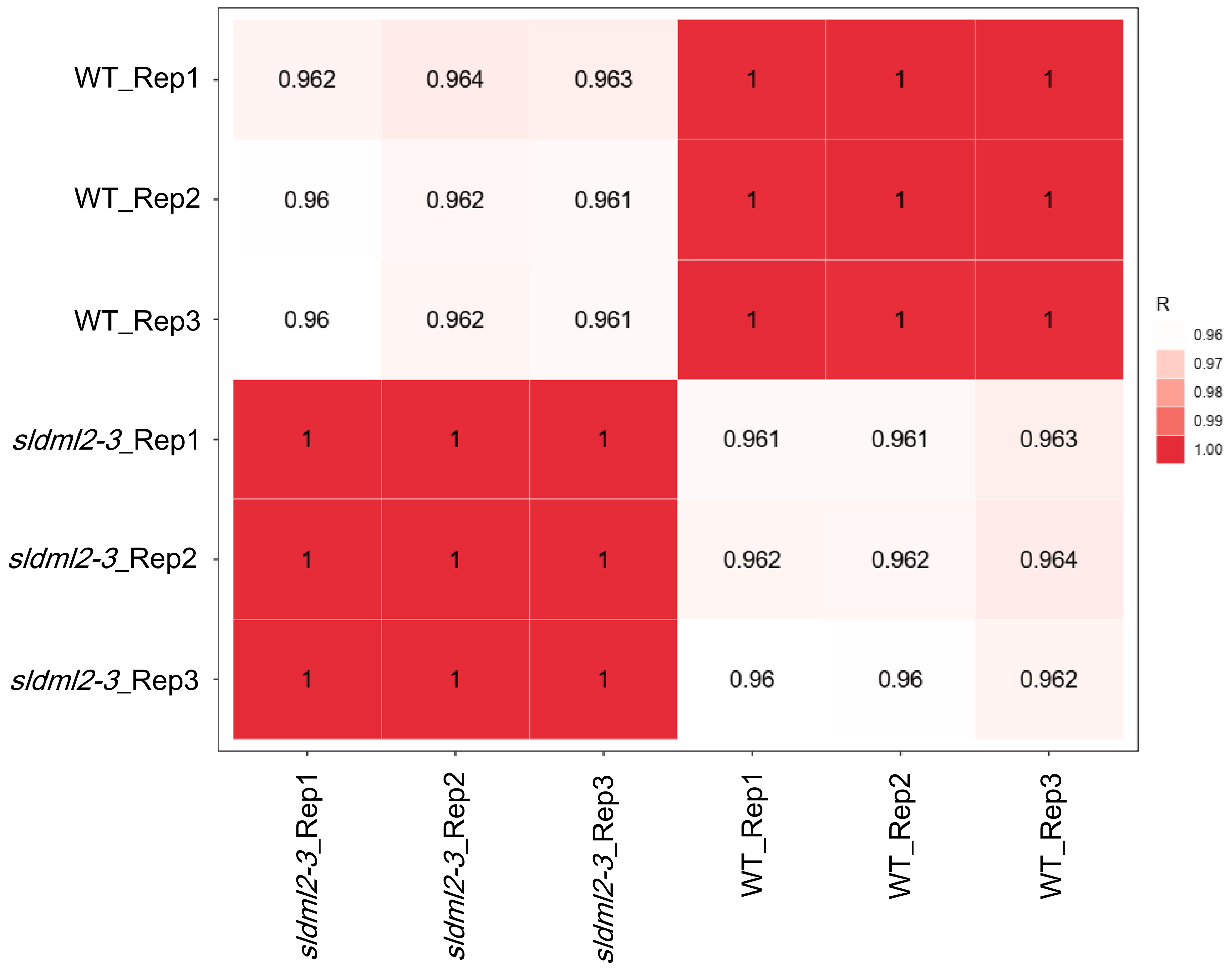

Figure S6 Pearson correlation coefficients between RNA-seq samples. RNA-seq was performed with three independent biological replicates in the wild-type (WT) and *sldml2-3* mutant fruits after inoculation with *B. cinerea* for two days. R, Pearson correlation coefficient; Rep, replicate.

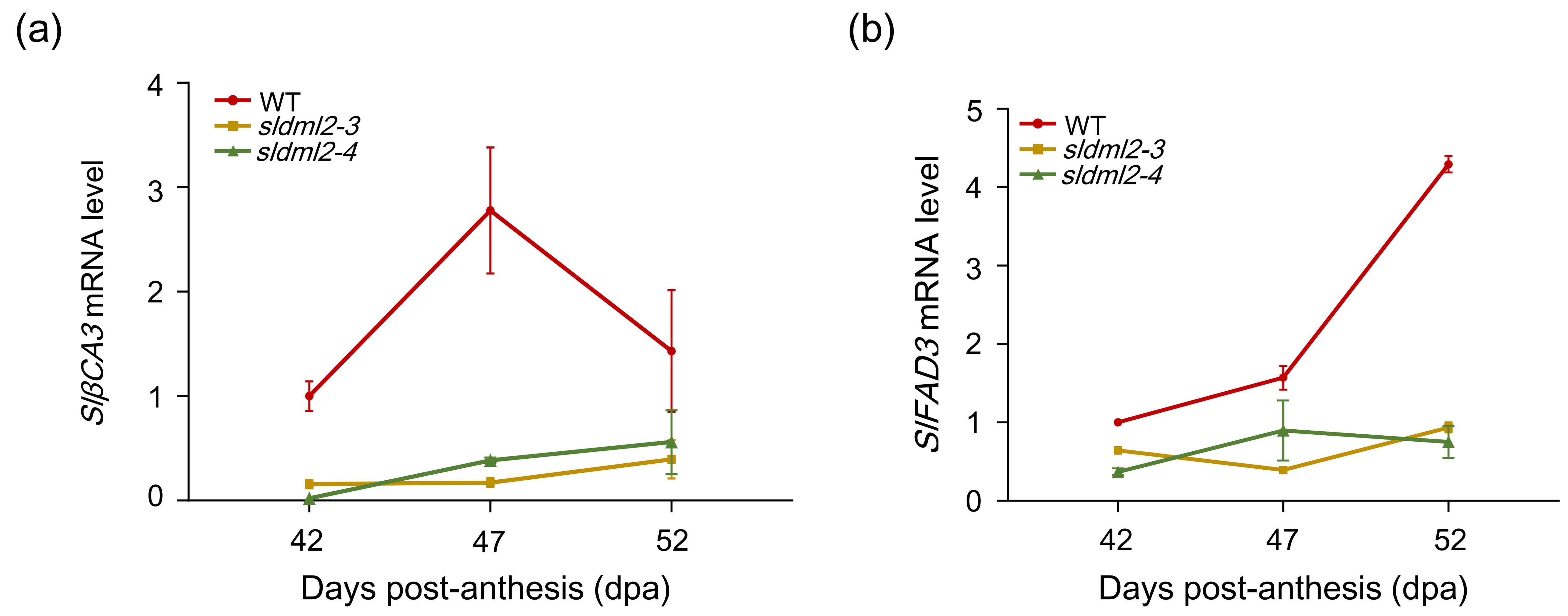

Figure S7 *SlDML2* mutation impairs the gene expression of *SlβCA3* and *SlFAD3*. (a) *SlβCA3* transcription level in fruits of the wild-type (WT), *sldml2-3*, and *sldml2-4* at 42, 47, and 52 dpa. (b) *SlFAD3* transcription level in fruits of the WT, *sldml2-3*, and *sldml2-4* at 42, 47, and 52 dpa. The tomato *SlUBI3* gene was used as an internal control. Data are presented as mean ± standard deviation (n = 3). dpa, days post-anthesis.

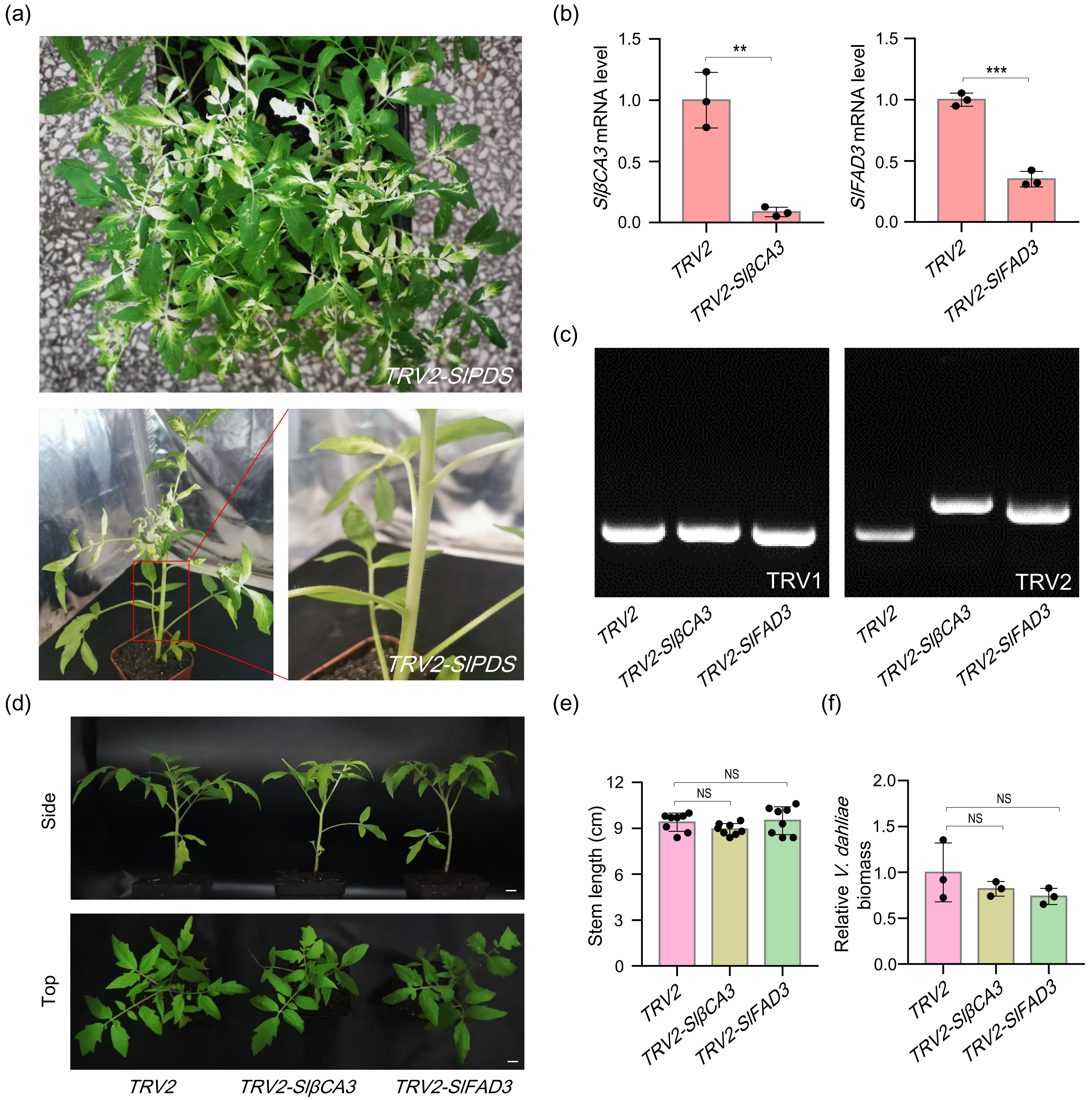

Figure S8 *SlβCA3* and *SlFAD3* show no effect on tomato resistance to *V. dahliae*. (a) Representative photographs of the *TRV2-SlPDS* seedlings exhibiting albinistic phenotypes in leaf and stem. The *TRV2-SlPDS* seedlings were used as a positive control for VIGS-mediated gene silence. *PDS*, *phytoene desaturase*. (b) Transcription levels of *SlβCA3* and *SlFAD3* in tomato stems after VIGS. Tomato seedlings infiltrated with the empty vector pTRV2 were used as the control group. The tomato *ACTIN* gene was used as an internal control. Asterisks indicate significant differences (***P* < 0.01, ****P* < 0.001; Student's t test). (c) PCR amplification showing that the virus vectors were successfully expressed in stems of the *TRV2*, *TRV2-SlβCA3*, and *TRV2-SlFAD3* seedlings. (d) Representative photographs and (e) stem lengths of the *TRV2*, *TRV2-SlβCA3*, and *TRV2-SlFAD3* seedlings that were inoculated with *V. dahliae* for 16 days. Scale bar = 1 cm. (f) Relative *V. dahliae* biomass in stems of the *TRV2*, *TRV2-SlβCA3*, and *TRV2-SlFAD3* seedlings that were inoculated with *V. dahliae* for 16 days. The amounts of *V. dahliae GADPH* gene were determined by quantitative RT-PCR analysis using the tomato *RuBisCo* gene as an internal control. NS, no significance.

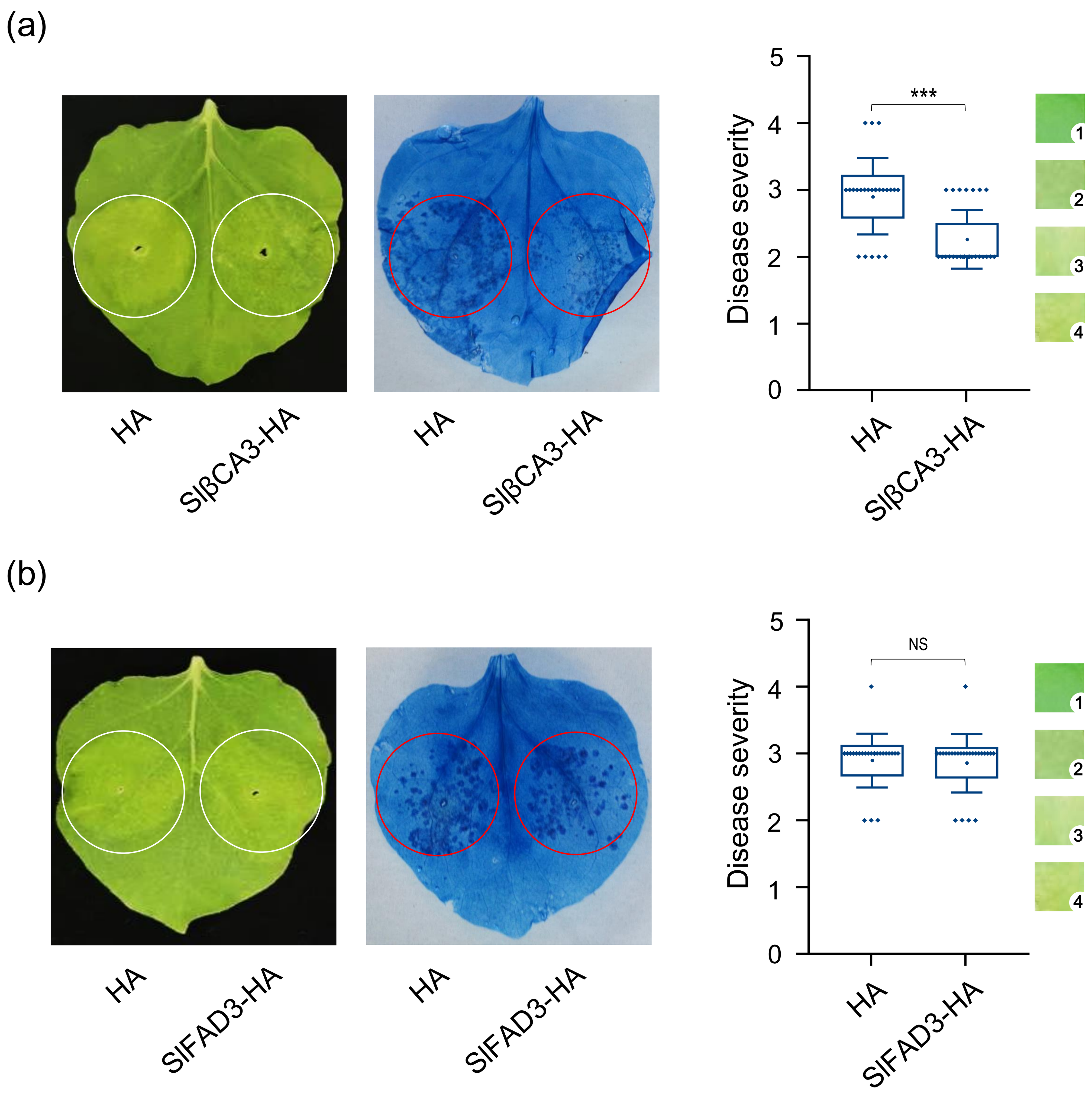

Figure S9 Roles of *SlβCA3* and *SlFAD3* in regulating resistance to *Pst* DC3000. (a) Disease symptoms and severity on *N. benthamiana* leaves with or without the expression of SlβCA3-HA fusion protein. (b) Disease symptoms and severity on *N. benthamiana* leaves with or without the expression of SlFAD3-HA fusion protein. Disease symptoms were observed after inoculation for five days, and the representative photographs of disease symptoms and trypan blue staining were shown. The number from 1 to 4 and the corresponding photographs of disease symptom represent increased degrees of disease severity. Asterisks indicate significant differences (****P* < 0.01; Student's t test). NS, no significance.

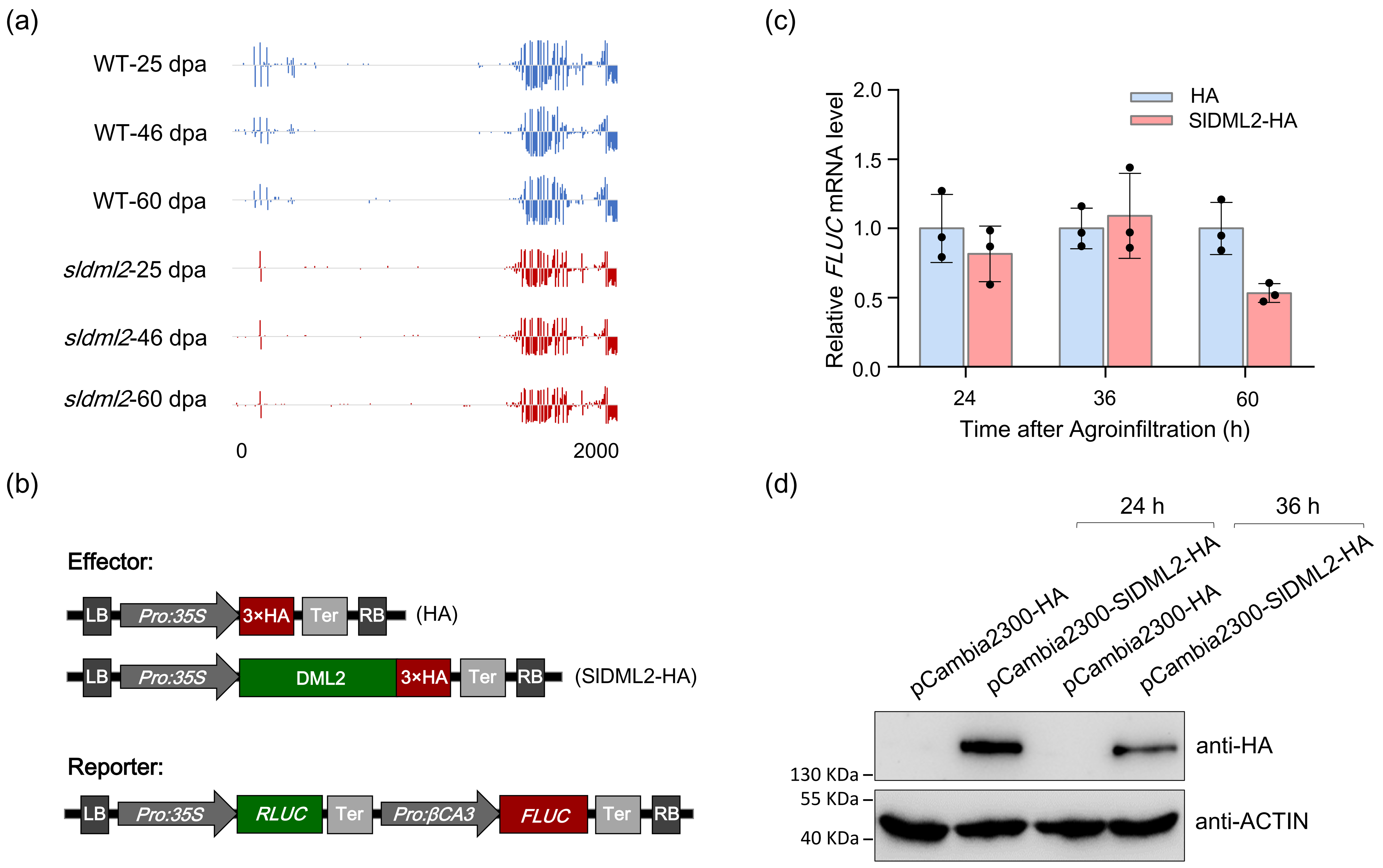

Figure S10 *SlDML2* mutation does not cause differential 5mC modification in the *SlβCA3* promoter. (a) DNA methylation (5mC) levels of the *SlβCA3* promoter in the wild-type (WT) and *sldml2* mutant fruits at indicated developmental stages. The 5mC level was analyzed by using the published DNA methylome database (Lang et al., 2017). Each vertical bar represents an 5mC and the bar height indicates 5mC level. dpa, days post-anthesis. (b) Schematic of the dual-luciferase system used for promoter activity assay. The *SlβCA3* promoter was cloned into the dual-luciferase reporter vector to drive the expression of *FLUC* gene. The *RLUC* gene driven by the CaMV 35S promoter served as an internal control. *FLUC*, *firefly luciferase*; *RLUC*, *renilla luciferase*; LB, left border; RB, right border; Ter, terminator. (c) Transcription levels of the *FLUC* gene derived by the *SlβCA3* promoter in *N. benthamiana* leaves with or without the co-expression of SlDML2-HA as determined by quantitative RT-PCR. The *RLUC* gene was used as an internal control. (d) Detection of expression of SlDML2-HA fusion protein in *N. benthamiana* leaves. Total protein was extracted at indicated time after agroinfiltration and then submitted to immunoblot with anti-HA antibody. Equal loading was confirmed by using the *N. benthamiana* ACTIN as an internal control.

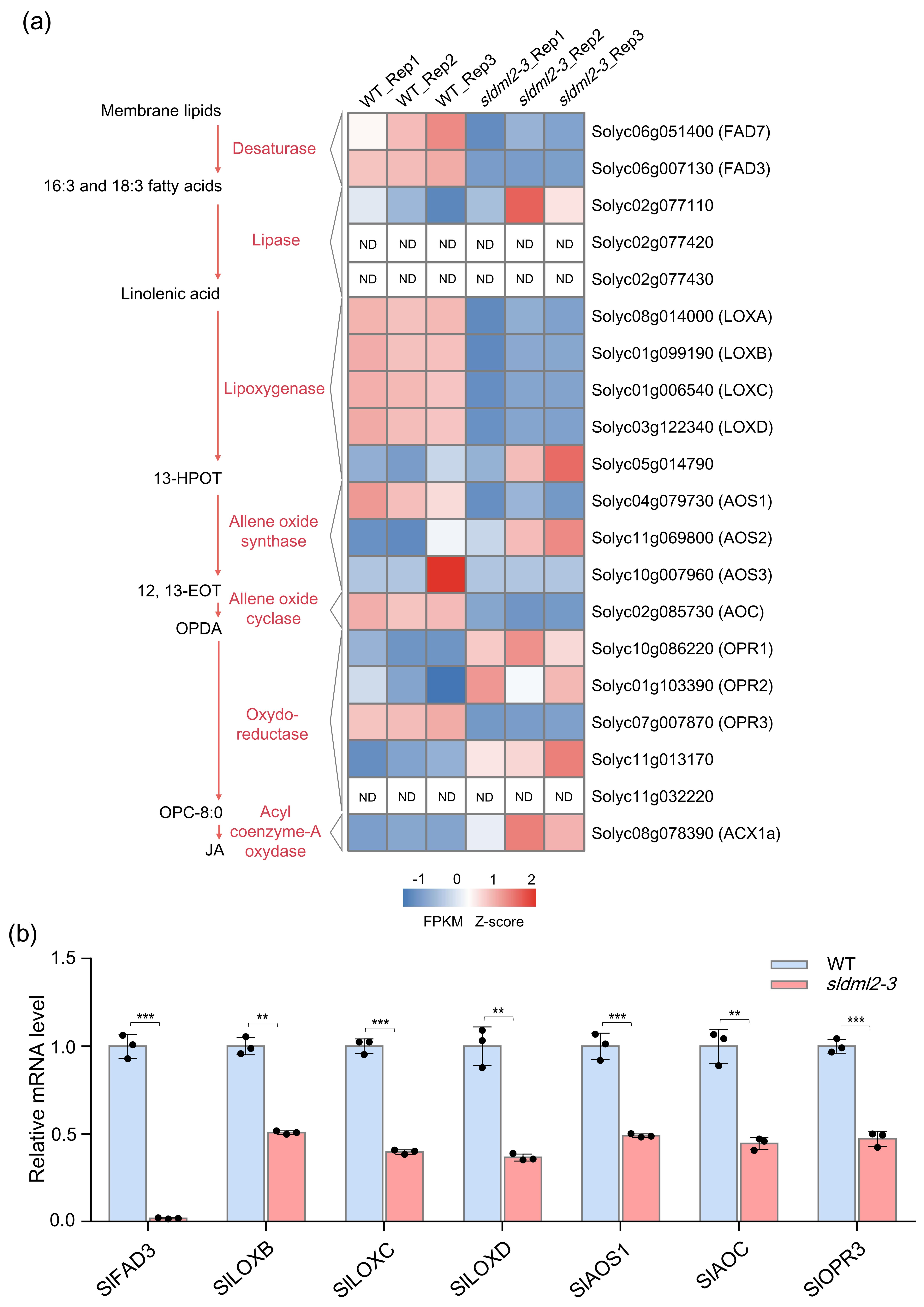

Figure S11 *SlDML2* mutation disturbs the expression of JA biosynthetic genes. (a) Heat map of transcription levels of JA biosynthetic genes in the wild-type (WT) and *sldml2-3* mutant fruits after *B. cinerea* inoculation for two days. The gene expression was determined by RNA-seq with three independent biological replicates. Rep, replicate; ND, not detected. (b) Validations of the transcription levels of several down-regulated JA biosynthetic genes in the *sldml2-3* mutant fruit compared to those of the WT by quantitative RT-PCR analysis. The tomato *SlUBI3* gene was used as an internal control. Asterisks indicate significant differences (** P < 0.01, *** P < 0.001; Student's t test). *LOXB*, *lipoxygenase B*; *LOXC*, *lipoxygenase C*; *LOXD*, *lipoxygenase D*; *AOS1*, *allene oxide synthase*; *AOC*, *allene oxide cyclase*; *OPR3*, *oxydoreductase 3*.

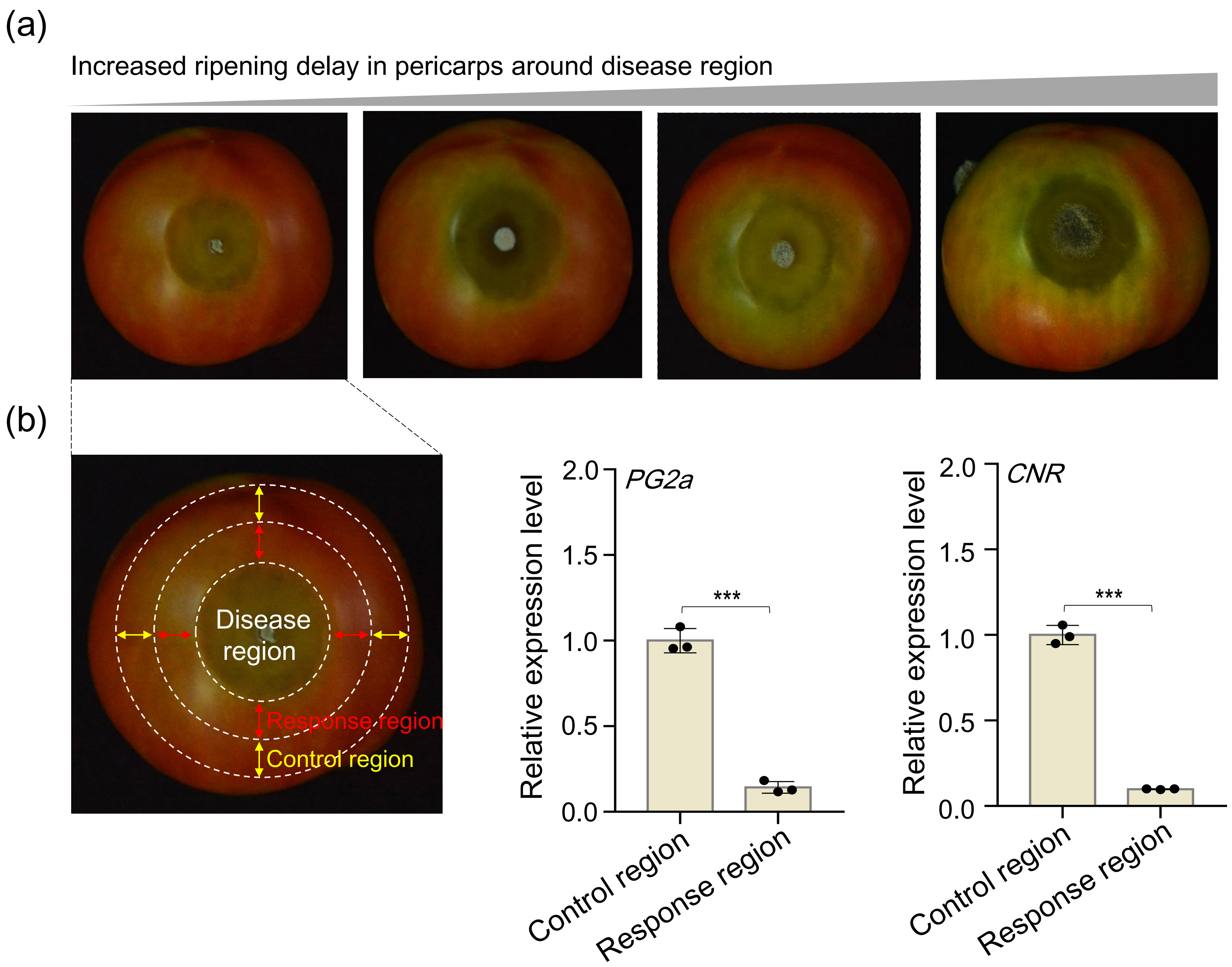

Figure S12 *B. cinerea* invasion causes a delay in ripening of tomato pericarp tissues around the disease region. (a) Photographs of wild-type tomato fruits with distinct ripening delay in pericarps around disease region after *B. cinerea* inoculation for four days. The pericarp tissue with a 5-mm thickness surrounding disease regions was defined as “response region”, and the pericarp tissue with a 5-mm thickness around the response region was defined as “control region”. (b) Transcription levels of ripening-related genes in the response region and control region as determined by quantitative RT-PCR analysis. The tomato *SlUBI3* gene was used as an internal control. *PG2a*, *polygalacturonase 2a*; *CNR*, *colorless nonripening*. Asterisks indicate significant differences (***$P < 0.001$; Student’s t test).

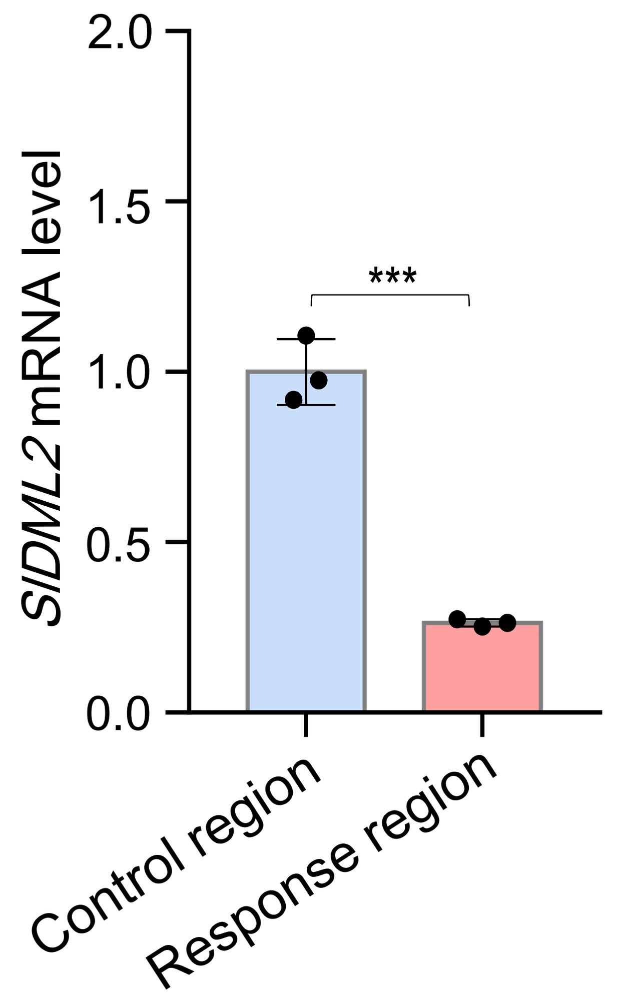

Figure S13 Influence of *B. cinerea* invasion on *SlDML2* gene expression. Transcription levels of *SlDML2* gene in the response region and control region were determined by quantitative RT-PCR analysis after *B. cinerea* inoculation for four days. The tomato *SlUBI3* gene was used as an internal control. Asterisks indicate significant differences (***$P < 0.001$; Student's t test).
