# Supplementary material for: The pivotal ripening gene SlDML2 participates in regulating disease resistance in tomato
Source: Plant Biotechnol J. 2023 Jul 19;21(11):2291–306. doi: 10.1111/pbi.14130 (PMC10579708; doi:10.1111/pbi.14130)
Supplement: Supplementary file 1 — Figure S1 Genotypes and predicted peptides of the SlDML2 locus in sldml2 mutants generated by CRISPR/Cas9‐mediated gene editing. Figure S2 Fruits of sldml2 mutants can turn red at the final ripe stage. Figure S3 SlDML2 mutation does not cause significant difference in seed appearance. Figure S4 SlDML2 gene expression in various tomato organs as determined by quantitative RT‐PCR. Figure S5 SlDML2 mutation does not cause significant difference in length of seedling stem. Figure S6 Pearson correlation coefficients between RNA‐seq samples. Figure S7 SlDML2 mutation impairs the gene expression of SlβCA3 and SlFAD3. Figure S8 SlβCA3 and SlFAD3 show no effect on tomato resistance to V. dahliae. Figure S9 Roles of SlβCA3 and SlFAD3 in regulating resistance to Pst DC3000. Figure S10 SlDML2 mutation does not cause differential 5mC modification in the SlβCA3 promoter. Figure S11 SlDML2 mutation disturbs the expression of JA biosynthetic genes. Figure S12 B. cinerea invasion causes a delay in ripening of tomato pericarp tissues around the disease region. Figure S13 Influence of B. cinerea invasion on SlDML2 gene expression. Method S1 V. dahliae culture condition and disease symptom assay. Method S2 Pst DC3000 culture condition and disease symptom assay. Method S3 5mC assay by bisulphite sequencing. Method S4 ChIP‐qPCR assay. Method S5 McrBC‐PCR assay. Method S6 Protein extraction and western blot. Method S7 VIGS. Method S8 Transient overexpression. Method S9 Lycopene content measurement. Method S10 Transcription activity assay. Method S11 Data analysis. Table S1 Up‐regulated genes in the fruit of sldml2‐3 mutant during the pathogenic process of B. cinerea compared with the wild‐type. Table S2 Down‐regulated genes in the fruit of sldml2‐3 mutant during the pathogenic process of B. cinerea compared with the wild‐type. Table S3 Gene ontology (GO) enrichment analysis of differentially expressed genes in the fruit of sldml2‐3 mutant during the pathogenic process of B. cinerea compar [file PBI-21-2291-s001.zip › pbi14130-sup-0002-Methods S1-S11.doc]

**Method S1**

***V. dahliae* culture condition and disease symptom assay**

*V. dahliae* strain V592 was cultured as the previously described (Feng et al., 2018). Briefly, *V. dahliae* was cultured on PDA medium at 26℃ in the dark for 3-5 days. The microsclerotia was then transferred to Czapek-Dox liquid medium (2 g·L^-1^ NaNO_3_, 1.32 g·L^-1^ K_2_HPO_4_·3H_2_O, 1 g·L^-1^ MgSO_4_·7H_2_O, 1 g·L^-1^ KCl, 0.01 g·L^-1^ FeSO_4_·7H_2_O, and 30 g·L^-1^ sucrose) for continue culture with shaking at 150 rpm for 5 days, 26℃. The conidiophores were purified by filtration through two layers of sterile gauze and diluted in sterile water to a final concentration of 1 × 10^7^ spores per millilitre. *V. dahliae* disease symptom assay was carried out according to the method of Fradin et al. (2009) with minor modification. Briefly, the roots of three-week-old tomato seedlings were rinsed with sterile water, and then dipped for 30 min in the prepared conidiophore suspension. Control seedlings (Mock) were treated similarly, but their roots were dipped in sterile water without conidiophores. The treated seedlings were subsequently replanted in fresh soil and cultured in a greenhouse. *V. dahliae* disease symptom was monitored by measurement of the stem length and the *V. dahliae* biomass at 2-3 weeks after inoculation. For the biomass quantification, the amounts of *V. dahliae* *GAPDH* gene in the stem above the cotyledons were relatively determined with RT-qPCR analysis by using the tomato *RuBisCo* gene as an internal control. Sequences of primers used for PCR amplification are listed in Table S5.

**Method S2**

***Pst DC3000* culture condition and disease symptom assay**

*Pst* DC3000 were cultured in King’s B liquid medium supplemented with 50 mg·L^-1^ rifampicin at 28℃ for 24-48 h. The bacterial cells were collected by centrifugation at 5,000 *g* for 5 min and then dissolved in 10 mM MgCl_2_ with 0.02% (v/v) Silwet-L77. For *Pst* DC3000 inoculation on tomato, the bacterial suspension at a OD_600_ of 0.1 was sprayed on whole leaves of four-week-old tomato seedlings. After inoculation for 3 days, disease symptom was assessed by trypan blue staining and quantification of bacterial leaf populations. For *Pst* DC3000 inoculation on *N. benthamiana*, the bacterial suspension at OD_600_ of 0.01 was injected into four-week-old *N. benthamiana* leaves using a 1 ml needleless syringe. After inoculation for 5 days, disease symptom was assessed by the trypan blue staining and the degree scoring of disease severity according to the previous method (Hu et al., 2020).

**Method S3**

**5mC assay by bisulfite sequencing**

DNA methylation (5mC) level in the DMR of *SlβCA3* and *SlFAD3* were assessed as previously described with minor modifications (Gao et al., 2012). Briefly, genomic DNA (gDNA) was extracted from fruits of the wild-type and *sldml2* mutants at 46 dpa using the Plant DNA extraction kit (Magen, D3187). Then, 400 ng of purified DNA was treated with bisulfite to produce mutations from cytosine (C) to thymine (T) using the EZ DNA methylation-gold kit (ZYMO Research, D5005). The double strands of mutated DNA (20 ng) were used as templates for PCR amplification, respectively, with specific-designed primers that do not containing cytosines or guanines as far as possible. The PCR products were purified and subsequently cloned into the pCE2 TA/Blunt-Zero vector (TSINGKE, C601-02), and at least 10 positive clones were detected by Sanger bisulfite sequencing. The 5mC level in each cytosine site was analyzed by calculating the ratio of C-T transformation. Sequences of primers used for PCR amplification are listed in Table S5.

**Method S4**

**ChIP-qPCR assay**

ChIP-qPCR assay was carried out as described previously with some modifications (Wang et al., 2021). Briefly, mixed pericarp tissues of at least ten fruits at 42 dpa were sliced and fixed in 1% (v/v) formaldehyde under vacuum for 10 min. The fixed materials were subjected to nuclear isolation according to the method of Wang et al. (2014). The isolated nuclei were sonicated to shear gDNA to an average size of approximately 500 bp. The materials were centrifugated at 12,000 *g* for 5 min, and the supernatant containing the sheared chromatin was collected. After being reverse cross-linked, 1/10 (v/v) of the supernatant was used as the input control, and the remaining was incubated with affinity-purified anti-SlDML2 antibody that were beforehand bound to ChIP^TM^ Protein A magnetic beads (Millipore, 16-663) for 8 h at 4°C. Then, the beads were washed successively with low-salt buffer, high-salt buffer, lithium chloride buffer, and TE buffer. The DNA-protein complexes were eluted from the beads by occasional rotation at 65°C for 30 min. The cross-linking between immunoprecipitated SlDML2 protein and DNA fragments was interrupted by adding 0.2 M NaCl and then incubating at 65°C for overnight. The immunoprecipitated DNA fragments were isolated using the Plant DNA extraction kit (Magen, D3187) and subsequently subjected to quantitative RT-PCR assay. Sequences of primers used for PCR amplification are listed in Table S5.

**Method S5**

**McrBC-PCR assay**

McrBC-PCR assay was performed as the described (Shinozaki et al., 2018) with minor modifications. Briefly, gDNA was extracted from tomato fruits or *N. benthamiana* leaves using the Plant DNA extraction kit (Magen, D3187), and quantified by a SimpliNano spectrophotometer (GE Healthcare, 29-0617-11). 400 ng gDNAs were digested with 4 U McrBC enzyme (NEB, M0272S) at 37°C for overnight, and the same reaction without the addition of GTP was concurrently performed as a negative control. PCR amplification was subsequently carried out with 20 ng of McrBC-treated DNA and the following program (50 μL): 95°C for 2 min, followed by 24-27 cycles of 95°C for 30 s, 55°C for 30 s, and 72°C for 1 min, and finally 72°C for 5 min. Sequences of primers used for PCR amplification are listed in Table S5.

**Method S6**

**Protein extraction and Western blot**

Total proteins were extracted using TCA acid lysis method as the described (Delaunay et al., 2000). Briefly, 100 mg of *N. benthamiana* leaves was powdered with liquid nitrogen and lysed in 400 µL of 12.5% (w/v) TCA. The lysate was centrifuged and the precipitated pellet was washed in acetone for twice. Then, the pellet was air-dried, dissolved in extracted buffer (100 mM Tris-HCl, pH 8.0, 1 mM EDTA, 1% (w/v) SDS, and 1 mM PMSF), and incubated at 25°C for 15 min. The extraction was centrifuged at 15000 *g* for 5 min and the supernatant was transferred into a new 1.5 mL centrifuge tube. Protein concentrations were analyzed by using the Pierce^TM^ BCA protein assay kit (Thermo Scientific, 23225). Protein samples were separated by 10% SDS-PAGE and electrotransferred onto PVDF membrane (Millipore, IPVH00010) by a Semi-dry transfer unit (Amersham, TE77). The membrane was blocked with 5% (w/v) non-fat milk in TBST buffer at room temperature for 2 h, and then incubated with primary anti-HA antibody (1:10000) or anti-ACTIN antibody (1:10000) at room temperature for another 2 h. After being washed four times with TBST buffer, the membrane was continually incubated with HRP-conjugated anti-rabbit IgG secondary antibody (1:10000) at room temperature for 1.5 h. Finally, the membrane was washed four times with TBST buffer and then visualized by using the Enhanced ECL chemiluminescence detection kit (Solarbio, PE0010).

**Method S7**

**VIGS**

Virus-induced gene silencing (VIGS) was carried out by using the virus vectors pTRV1 and pTRV2 according to the method of Meng et al. (2016). Briefly, the specific cDNA fragments of *SlβCA3* and *SlFAD3* were designed by the VIGS tool from the Sol Genomics Network (https://solgenomics.net/), and then cloned into the pTRV2 vector. The constructed pTRV2-*SlβCA3* and pTRV2-*SlFAD3* vectors, as well as the pTRV1 vector, were transformed into *A. tumefaciens* strain GV3101. The *Agrobacterium* were grown at 28°C for 24 h in LB liquid medium supplemented with 50 μg·ml^-1^ kanamycin, 50 μg·ml^-1^ gentamycin, and 50 μg·ml^-1^ rifampicin. Then, the bacterial cells were harvested and resuspended in the infiltration medium (10 mM MES pH 5.6, 10 mM MgCl_2_, 100 µM acetosyringone) to a final OD_600nm_ of 0.5. The *Agrobacterium* containing pTRV1 and the *Agrobacterium* harboring pTRV2, pTRV2-*SlβCA3*, or pTRV2-*SlFAD3* were mixed at a ratio of 1:1 (v/v) and injected into the cotyledons of one-week-old tomato seedlings with a 1 ml needleless syringe. The infected seedlings were subsequently cultured in a greenhouse with 22°C, 60%-80% relative humidity, and a 12/12 h light/dark photoperiod. The *phytoene desaturase* (*PDS*) gene was used as the positive control. Sequences of primers used for the construction of pTRV2-*SlβCA3* and pTRV2-*SlFAD3* vectors are listed in Table S5.

**Method S8**

**Transient overexpression**

The coding sequence of *SlβCA3* and *SlFAD3* were cloned from tomato cDNAs and inserted into the pCambia1302-MCS-3HA vector under the drive of CaMV 35S promoter. The constructed vectors were transformed into *A. tumefaciens* strain GV3101, and then grown at 28°C for overnight in LB liquid medium supplemented with 50 μg·ml^-1^ kanamycin, 50 μg·ml^-1^ gentamycin, and 50 μg·ml^-1^ rifampicin. The bacterial cells were harvested and resuspended in the infiltration medium (10 mM MES pH 5.6, 10 mM MgCl_2_, 100 µM acetosyringone) to a final OD_600nm_ of 0.5. The medium was injected into the true leaves of three to four-week-old wild-type or *sldml2* mutant seedlings using a 1 ml needleless syringe. After 36 h, total proteins were extracted and analyzed by western blot as described above.

**Method S9**

**Lycopene content measurement**

Lycopene content was measured as the described (Sun et al., 2015) with minor modifications. Briefly, 0.2 g of tomato pericarp tissues was powdered with liquid nitrogen and lysed in 2 mL of hexane-acetone-ethanol (2:1:1, v/v). The lysate was homogenized for 1 min by vortex. After homogenization, 0.6 mL of ddH_2_O was added and the samples were vortexed for 10 s. Following phase separation on ice for 5 min, the upper organic phase was collected and the absorbance at 503 nm was measured. Lycopene content was calculated with a unit of mg kg^-1^ by using the method of Fish et al (2002).

**Method S10**

**Transcription activity assay**

The transcription activity of *SlβCA3* and *SlFAD3* promoters was analyzed by using the dual-luciferase reporter system as the described (Han et al., 2016). In brief, the sequence fragments of *SlβCA3* and *SlFAD3* promoters (~2000 bp upstream of the start codon) were respectively amplified and inserted into the dual-luciferase reporter vector pGreen II-0800-LUC to drive the expression of *Firefly luciferase* (*FLUC*) reporter gene. To analyze the transcription activity of *SlβCA3* promoter to its own transcript, the *SlβCA3* gene body containing all exons and introns was cloned and then inserted into the dual-luciferase reporter vector with a replacement with the *FLUC* gene. The coding sequence of *SlDML2* was cloned into the pCambia2300-MCS-HA vector driven by the 35S promoter for the expression of SlDML2 protein that serves as an effector. The constructs were transformed into *Agrobacterium tumefaciens* strain GV3101 that contains the pSoup vector. The *Agrobacterium* was cultured in LB liquid medium containing 50 μg mL^-1^ kanamycin, 50 μg mL^-1^ gentamycin and 50 μg ml^-1^ rifampicin at 28°C for 24 h. Then, the *Agrobacterium* was resuspended in the infiltration medium (10 mM MES, pH 5.6, 10 mM MgCl_2_, and 100 µM acetosyringone) to a final OD_600_ of 0.4. The *Agrobacterium* harboring the reporter vector was co-transformed into *N. benthamiana* leaves with the *Agrobacterium* carrying the effector vector at a 1:1 ratio. At 36 h after agroinfiltration, the *N. benthamiana* leaves were collected and the activity of cytosol-synthesized FLUC was visualized by chemiluminescence with pseudo-colour and quantitatively analyzed using the Dual luciferase assay kit (Promega, E1910). The transcription level of *FLUC* or *SlβCA3* gene was detected by quantitative RT-PCR assay. The *Renilla luciferase* (*RLUC*) gene driven by the CaMV 35S promoter was utilized as the internal control. Sequences of primers used for vector constructions and quantitative RT-PCR analysis are listed in Table S5.

**Method S11**

**Data analysis**

GraphPad Prism 7.00 and Microsoft Excel 2013 were used for data analyses. Comparative analysis was carried out by two-tailed Student’s t-test, and statistically significant differences were indicated by asterisks (**P* < 0.05, ***P* < 0.01, ****P* < 0.001).

**References**

Delaunay, A., Isnard, A.D. and Toledano, M.B. (2000) H_2_O_2_ sensing through oxidation of the Yap1 transcription factor. *EMBO J* **19**, 5157-5166.

Feng, Z., Tian, J., Han, L., Geng, Y., Sun, J. and Kong Z.S. (2018) The Myosin5-mediated actomyosin motility system is required for Verticillium pathogenesis of cotton. *Environ Microbiol* **20**, 1607-1621.

Fish, W.W., Perkins-Veazie, P. and Collins, J.K. (2002) A quantitative assay for lycopene that utilizes reduced volumes of organic solvents. *J Food Compos Anal* **15**, 309-317.

Fradin, E.F., Zhang, Z., Juarez Ayala, J.C., Castroverde, C.D., Nazar, R.N., Robb, J., Liu, C. and Thomma, B.P. (2009) Genetic dissection of Verticillium wilt resistance mediated by tomato Ve1. *Plant Physiol* **150**, 320-332.

Gao, F., Liu, X., Wu, X., Wang, X., Gong, D., Lu, H., Xia, Y., Song, Y., Wang, J., Du, J., Liu, S., Han, X., Tang, Y., Yang, H., Jin, Q., Zhang, X. and Liu, M. (2012) Differential DNA methylation in discrete developmental stages of the parasitic nematode *Trichinella spiralis*. *Genome Biol* **13**, R100.

Han, Y., Kuang, J., Chen, J., Liu, X., Xiao, Y., Fu, C., Wang, J., Wu, K. and Lu, W. (2016) Banana transcription factor MaERF11 recruits histone deacetylase MaHDA1 and represses the expression of *MaACO1* and expansins during fruit ripening. *Plant Physiol* **171**, 1070-1084.

Hu, Z., Ma, Q., Foyer, C.H., Lei, C., Choi, H.W., Zheng, C., Li, J., Zuo, J., Mao, Z., Mei, Y., Yu, J., Klessig, D.F. and Shi, K. (2021) High CO_2_- and pathogen-driven expression of the carbonic anhydrase βCA3 confers basal immunity in tomato. *New Phytol* **229**, 2827-2843.

Meng, L., Wang, R., Zhu, B., Zhu, H., Luo, Y. and Fu, D.Q. (2016) Efficient virus-induced gene silencing in *Solanum rostratum*. *PLoS One* **11**, e0156228.

Shinozaki, Y., Nicolas, P., Fernandez-Pozo, N., Ma, Q., Evanich, D.J., Shi, Y., Xu, Y., Zheng, Y., Snyder, S.I., Martin, L.B.B., Ruiz-May, E., Thannhauser, T.W., Chen, K., Domozych, D.S., Catala, C., Fei, Z., Mueller, L.A., Giovannoni, J.J. and Rose, J.K.C. (2018) High-resolution spatiotemporal transcriptome mapping of tomato fruit development and ripening. *Nat Commun* **9**, 364.

Sun, Q., Zhang, N., Wang, J., Zhang, H., Li, D., Shi, J., Li, R., Weeda, S., Zhao, B., Ren, S. and Guo, Y. (2015) Melatonin promotes ripening and improves quality of tomato fruit during postharvest life. *J Exp Bot* **66**, 657-668.

Wang, W., Wang, P., Li, X., Wang, Y., Tian, S. and Qin G. (2021) The transcription factor SlHY5 regulates the ripening of tomato fruit at both the transcriptional and translational levels. *Hortic Res* **8**, 83.

Wang, Y,, Wang, W., Cai, J., Zhang, Y., Qin, G. and Tian, S. (2014) Tomato nuclear proteome reveals the involvement of specific E2 ubiquitin-conjugating enzymes in fruit ripening. *Genome Biol* **15**, 548.
